# Slow-Onset Inhibition of *Mycobacterium tuberculosis* InhA: Revealing Molecular Determinants of Residence Time by MD Simulations

**DOI:** 10.1371/journal.pone.0127009

**Published:** 2015-05-21

**Authors:** Benjamin Merget, Christoph A. Sotriffer

**Affiliations:** Institute of Pharmacy and Food Chemistry, University of Würzburg, Am Hubland, D-97074, Würzburg, Germany; Oak Ridge National Laboratory, UNITED STATES

## Abstract

An important kinetic parameter for drug efficacy is the residence time of a compound at a drug target, which is related to the dissociation rate constant *k_off_*. For the essential antimycobacterial target InhA, this parameter is most likely governed by the ordering of the flexible substrate binding loop (SBL). Whereas the diphenyl ether inhibitors **6PP** and triclosan (**TCL**) do not show loop ordering and thus, no slow-binding inhibition and high *k_off_* values, the slightly modified **PT70** leads to an ordered loop and a residence time of 24 minutes. To assess the structural differences of the complexes from a dynamic point of view, molecular dynamics (MD) simulations with a total sampling time of 3.0 *µ*s were performed for three ligand-bound and two ligand-free (perturbed) InhA systems. The individual simulations show comparable conformational features with respect to both the binding pocket and the SBL, allowing to define five recurring conformational families. Based on their different occurrence frequencies in the simulated systems, the conformational preferences could be linked to structural differences of the respective ligands to reveal important determinants of residence time. The most abundant conformation besides the stable EI* state is characterized by a shift of Ile202 and Val203 toward the hydrophobic pocket of InhA. The analyses revealed potential directions for avoiding this conformational change and, thus, hindering rapid dissociation: (1) an anchor group in 2'-position of the B-ring for scaffold stabilization, (2) proper occupation of the hydrophobic pocket, and (3) the introduction of a barricade substituent in 5'-position of the diphenyl ether B-ring.

## Introduction

Although the death rate has dropped by 45% over the past two decades, tuberculosis (TB) is still a globally present disease. In 2012, 8.6 million new infections were documented and 1.3 million ended lethally [1]. The classical antitubercular therapy—based primarily on cocktails of isoniazid, rifampicin, pyrazinamide, and ethambutol for a period of six months—has cured over 56 million people since 1995, but the emergence of multi- and extensively drug-resistant strains of *Mycobacterium tuberculosis* (MDR-TB and XDR-TB) demands new, high-affinity inhibitor classes, which are unaffected by mycobacterial resistances [1–3].

Diphenyl ethers are one class of inhibitors currently under investigation. They bind directly to the well validated mycobacterial drug target enoyl-ACP reductase (InhA) without the necessity for prior activation by the enzyme catalase-peroxidase (KatG) [3]. InhA-inhibitors target the fatty acid synthesis II (FASII) of mycobacteria by disabling the hydrogenation of the unsaturated precursors of the long and hydrophobic mycolic acids, which are necessary for proper construction of the largely impermeable *Mycobacterium tuberculosis* (*Mtb*) cell wall [4].

**Fig 1:**
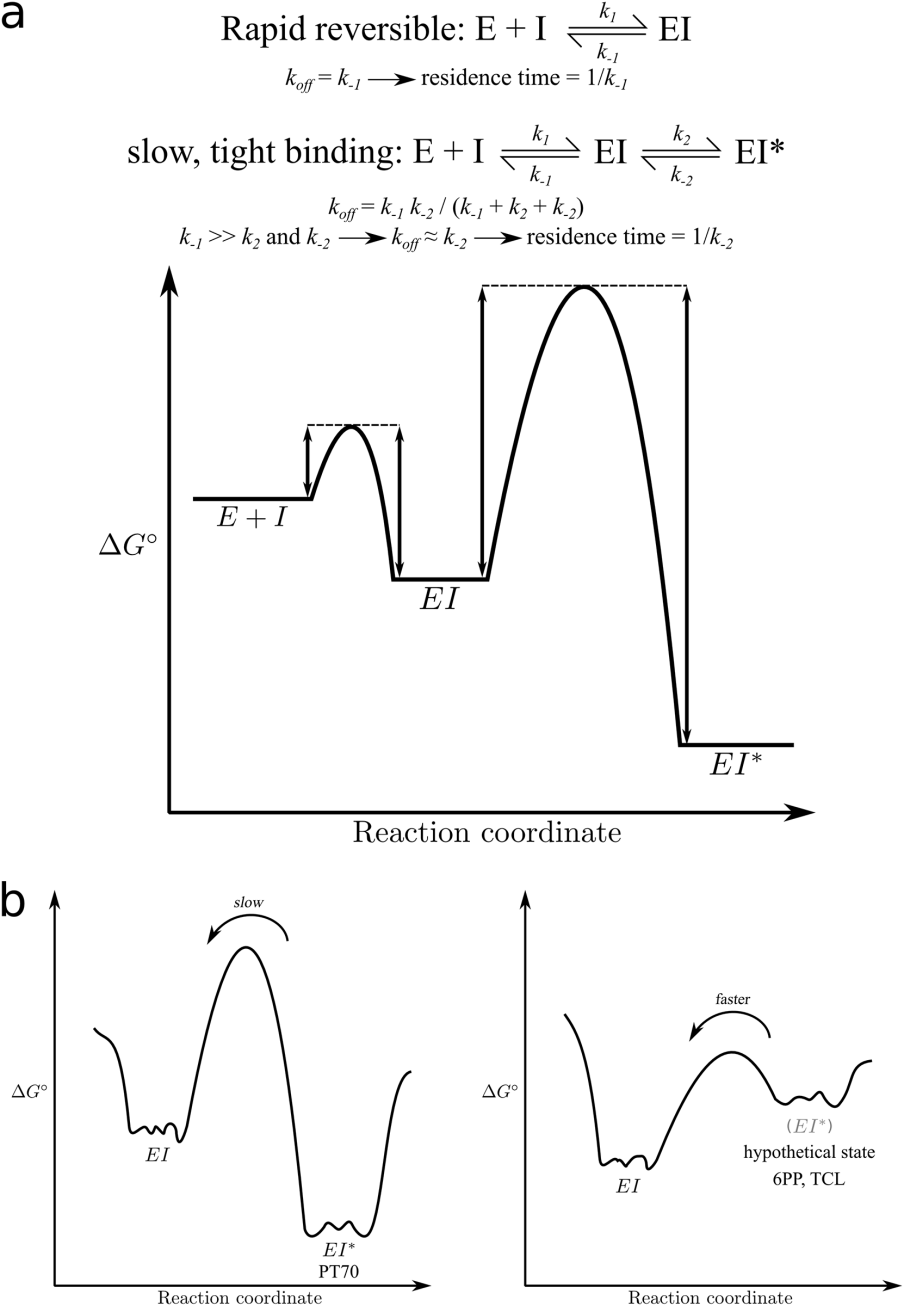
Mechanisms of drug-target complex formation. **(a)** Equilibria for rapid reversible inhibition via a one-step mechanism and slow-binding inhibition via a two-step induced-fit mechanism along with a schematic free-energy profile for this reaction. E denotes the enzyme, I the inhibitor, EI the initial enzyme-inhibitor complex, and EI* the final enzyme-inhibitor complex. In the case of InhA, the diphenyl ether inhibitors bind to the enzyme with bound oxidized cofactor NAD^+^, forming a ternary complex. The double-headed arrows in the energy profile highlight the importance of the barrier height for the kinetics of the reaction. **(b)** Schematic free-energy profiles for a slow-binding inhibitor (left) and a destabilized EI* state as a consequence of ligand removal or the presence of a rapid-reversible inhibitor (right). Each macrostate (EI, EI*) is obviously associated with many microstates.

To obtain highly active inhibitors, projects in early drug discovery generally focus on optimizing the affinity of candidate compounds for a given target. However, even for high-affinity inhibitors with *K*_*i*_ or *K*_*d*_ values in the low nanomolar range there is a potential activity gap between the *in vitro* assay experiments and a realistic *in vivo* system, where the exposure of target enzymes to drug-like molecules and the subsequent binding event can no longer be correctly described by equilibrium constants like *K*_*d*_. Rather, the dissociation rate constant (*k*_*off*_) of a protein-ligand complex, the reciprocal value of which describes the residence time (*t*_*R*_) of a compound at a drug target, should be considered during rational drug-design endeavors [5]. Thus, to reduce dosage and increase efficacy, it is desirable to optimize potential drugs in terms of a long residence time (i.e., low *k*_*off*_). Inhibitors exhibiting such low dissociation (and/or association) rate constants are termed “slow-onset inhibitors”, “slow-binding inhibitors” or briefly “slow-binders”. Although several different kinetic mechanisms are described for slow-binders, most of these inhibitors bind via an induced-fit mechanism [6]. The first initial complex (EI) is formed rapidly, whereupon a slower conformational change of the receptor allows the ligand to form the final complex (EI*) (Fig 1). For such slow-binding ligands, *k*_*off*_ is a combination of multiple individual rate constants. In detail, *k*_*off*_ can be described by *k*_-1_ ⋅ *k*_-2_ divided by (*k*_-1_ + *k*_2_ + *k*_-2_); if *k*_-1_ is large compared to *k*_2_ and *k*_-2_, *k*_*off*_ is essentially given by *k*_-2_ [6].

**Fig 2:**
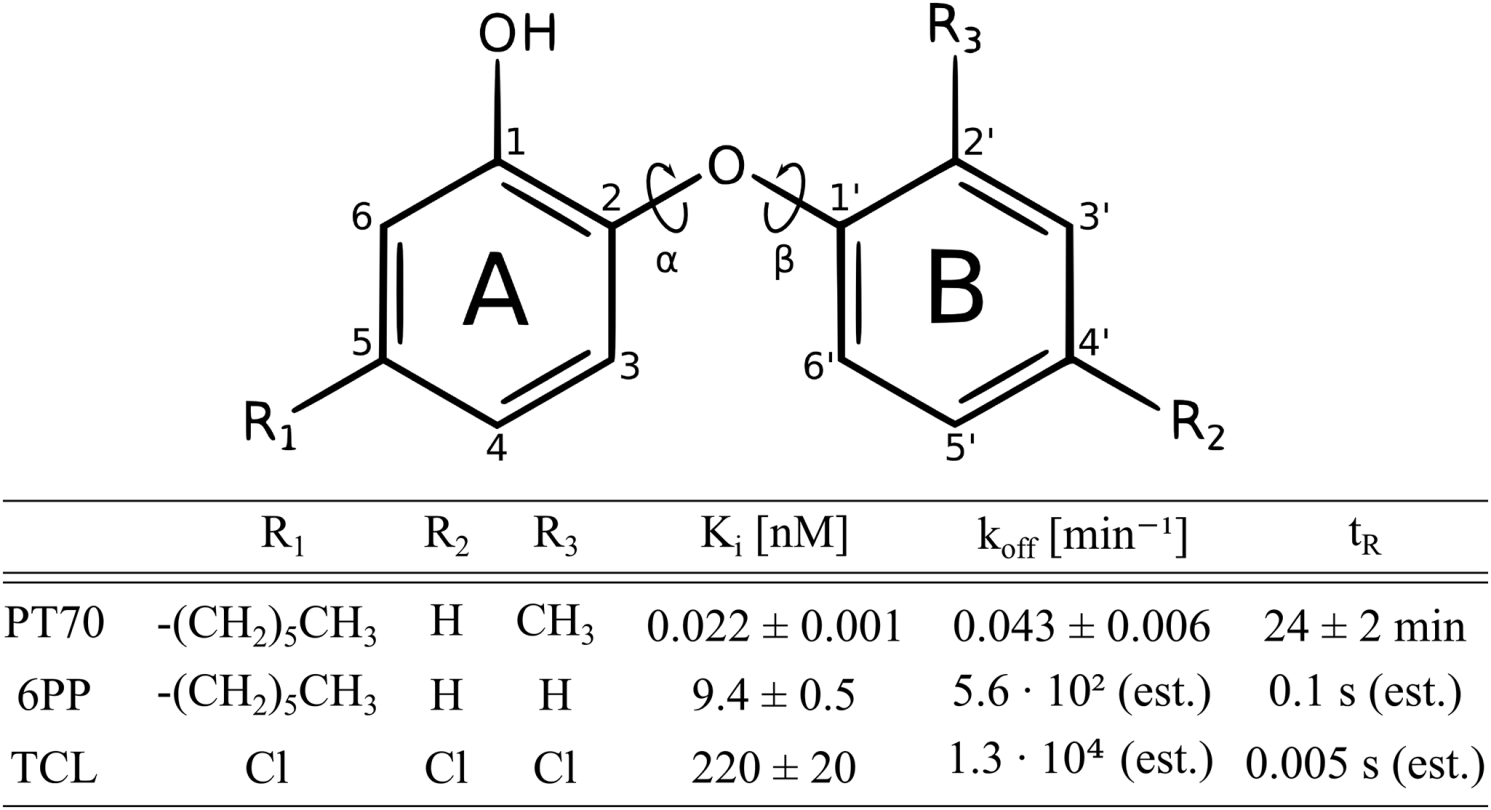
Overview of the experimentally characterized diphenyl ethers analyzed in the MD simulations. The phenyl rings are referred to as A- and B-ring, respectively. The corresponding ether torsions are symbolized by curly arrows and labeled *α* and *β*. Experimental data were taken from [7] and references provided therein. **PT70** is a slow-onset inhibitor, with measured dissociation rate constant *k*_*off*_ and residence time *t*_*r*_. In contrast, **6PP** and **TCL** show rapid-reversible binding kinetics; *k*_*off*_ and *t*_*r*_ values were estimated assuming a value of 10^9^ M^−1^s^−1^ for *k*_*on*_, as in [7].

Among the antitubercular diphenyl ethers, **PT70** displays slow-binding inhibition of InhA with a residence time of 24 minutes [7]. The broad-spectrum biocide triclosan (**TCL**), however, shows a rapid reversible inhibition of *Mtb* InhA, although it is a slow-binder in homologous enoyl-ACP reductases [8–13]. In InhA, slow-binding inhibition is likely associated with the ordering of the substrate binding loop (SBL, formed by helices *α*6 and *α*7), which is the most flexible region of InhA [7, 14]. In fact, the crystal structure of the InhA-NAD^+^-**PT70** complex (PDB code 2X23) shows an uninterrupted and highly ordered SBL, whereas in the crystal structure of the InhA-NAD^+^-**TCL** complex (PDB code 2B35) the SBL is unresolved due to high mobility [6, 13]. Thus, the highly ordered loop conformation very likely represents the final stage of the two-step binding mechanism (EI*) of the slow-binding inhibitor **PT70**. Although these observations are experimentally well characterized, it remains unclear how the structural features of a ligand govern the binding mechanism and, hence, the actual residence time. Knowing these features is essential for rationally modulating the residence time as a key parameter in drug design, even more so as small differences in the ligand structure can dramatically affect the dissociation rate constant. Besides **PT70** and **TCL**, the diphenyl ether **6PP** can serve as an illustrative example: it differs from **PT70** by only a methyl group, but nevertheless shows rapid reversible instead of slow tight binding behavior [3, 13, 15]. Fig 2 provides an overview of the investigated ligands and the associated experimental data.

As loop ordering and related conformational changes upon ligand binding are the most likely key factors in this context, we have conducted an extensive computational survey to elucidate the effects of different ligand structures on InhA conformational dynamics by means of molecular dynamics (MD) simulations. To this aim, five systems were prepared for simulation: (1) the unmodified InhA crystal structure with bound **PT70** and NAD^+^ (PDB code 2X23) [7], (2) the same crystal structure without inhibitor (i.e., after removing it; hereinafter called *perturbed*), and (3) the same crystal structure without ligand and cofactor (hereinafter called *No NAD^+^*). Furthermore, based again on PDB structure 2X23, complexes of InhA with NAD^+^ and the rapid reversible inhibitors (4) triclosan (**TCL**) and (5) **6PP** were setup. By starting all simulations from the highly ordered 2X23 crystal structure, it is possible to analyze perturbation effects and to reverse-engineer the potential EI*-complex formation. Placing **TCL** or **6PP** in the closed-SBL conformation of 2X23 enables the simulation of the virtual EI*-state of an InhA-NAD^+^-**TCL** or -**6PP** complex and examination of the dynamic properties that might eventually lead to loop disordering. With simulations based on these systems, we aim at revealing features of the conformational dynamics of the binding pocket and the SBL of InhA while linking them to structural differences in the respective ligands. Understanding the benefits and disadvantages of ligand properties in this context has implications for inhibitor design and optimization toward a longer residence time.

## Results and Discussion

This study is based on three major hypotheses: (1) The ternary complex of **PT70** with InhA and NAD^+^ represents the EI* state of the system. According to the current literature, there is no doubt about the validity of this assumption [7, 16, 17]. (2) As recently suggested by Li et al. [17], the EI state most likely corresponds to the open conformation of helix *α*6 (SBL) with respect to the binding pocket. This open conformation is observed, for example, in a substrate-analogue complex of InhA (PDB code 1BVR [18]). In contrast, the EI* state seen in the **PT70**-complex is characterized by a closed conformation of helix *α*6. (3) In the presence of inhibitors with rapid reversible binding kinetics, the EI* state is destabilized relative to the EI state. Therefore, after association of such an inhibitor, the EI* state is not reached, at least not to an observable extent. Conversely, placing a rapid reversible inhibitor in an EI* structure should cause its destabilization and eventually lead to the EI state. While experimentally hardly accessible, such a process can be investigated computationally. As illustrated in the schematic free energy profile of Fig 1b, destabilization of the EI* state in the presence of a rapid reversible inhibitor (or in the absence of an inhibitor) may lower the barrier to such an extent that a transition from EI* to EI could become observable within the time scale of standard unbiased MD simulations. This is the rationale for setting up the simulations with the inhibitors **6PP** and **TCL** placed in the binding pocket of the **PT70**-InhA crystal structure 2X23. The question then is whether and to which extent the EI* state is left under such conditions, whether an EI state is indeed reached and how all of this depends on the nature of the ligand.

In light of these hypotheses and questions, the outline for the analysis of the trajectories and the presentation of the results is as follows: We first focus on the binding pocket dynamics and examine the conformations observed in the **6PP**- and **TCL**-bound systems in comparison to the **PT70**-complex. To this aim, we perform a hierarchical cluster analysis on the basis of 2D-RMSD data of the three trajectories to reveal the conformational families visited by the simulations. This is followed by a closer analysis of the dynamics of the SBL, as well as of the ligand binding modes and the hydrogen-bond interactions. We attempt to link the observations in the different complexes to differences in the ligands, examining in particular the effect of the *ortho*-methyl substitution of **PT70**. We finally discuss the conformational families in the context of available experimental information, especially with respect to the presumed EI and EI* states. We conclude with a discussion of the implications for drug design and rational residence time modulation.

Because InhA crystallizes as a homotetramer and is known to be active as a homotetramer in solution [18], all simulations were run for the tetramer to best represent the bioactive form of InhA. This has the additional advantage of simultaneously sampling four analogous subunits at the same time. As the active sites of the four monomers are about 40 Å apart from each other, facing opposite sides in the quaternary structure and working independently [19], the 150 ns trajectories of the four binding pockets may be seen as a combined 600 ns sampling for the monomer. In a dynamic cross-correlation analysis of the four binding pockets over the entire trajectory we could not observe any correlated motions among the four pockets, supporting the assumption that their movements can be treated as independent. Therefore, in some of the analyses presented below, the combined ensembles of the four monomers were used. In other cases, however, it was more appropriate to follow the monomers individually along their 150 ns trajectory.

### Binding pocket dynamics and conformational families

We first focus on the binding pocket and compare the conformations observed in the different simulations to identify distinct conformational states, viz. recurring conformational families. The InhA binding pocket as defined by Luckner *et al.* (2010) [7] comprises the amino acids Phe149, Ala198, Met199, Ile202, and Val203 of the hydrophobic pocket, as well as the more hydrophilic residue Tyr158, which is an important hydrogen-bonding interaction partner for inhibitors. To detect conformational families of the ligand-bound state of the binding pocket, a 12x12 2D-RMSD plot of all against all monomers of the **PT70**-, **TCL**-, and **6PP**-complexes was calculated (see Supporting Information S1 Fig). This allows us to compare all conformations occurring in the different simulations and to identify similarities or differences across the systems, which is done most straightforwardly by a hierarchical cluster analysis on the basis of this 2D-RMSD matrix to group the recurring conformations to conformational families.

**Fig 3:**
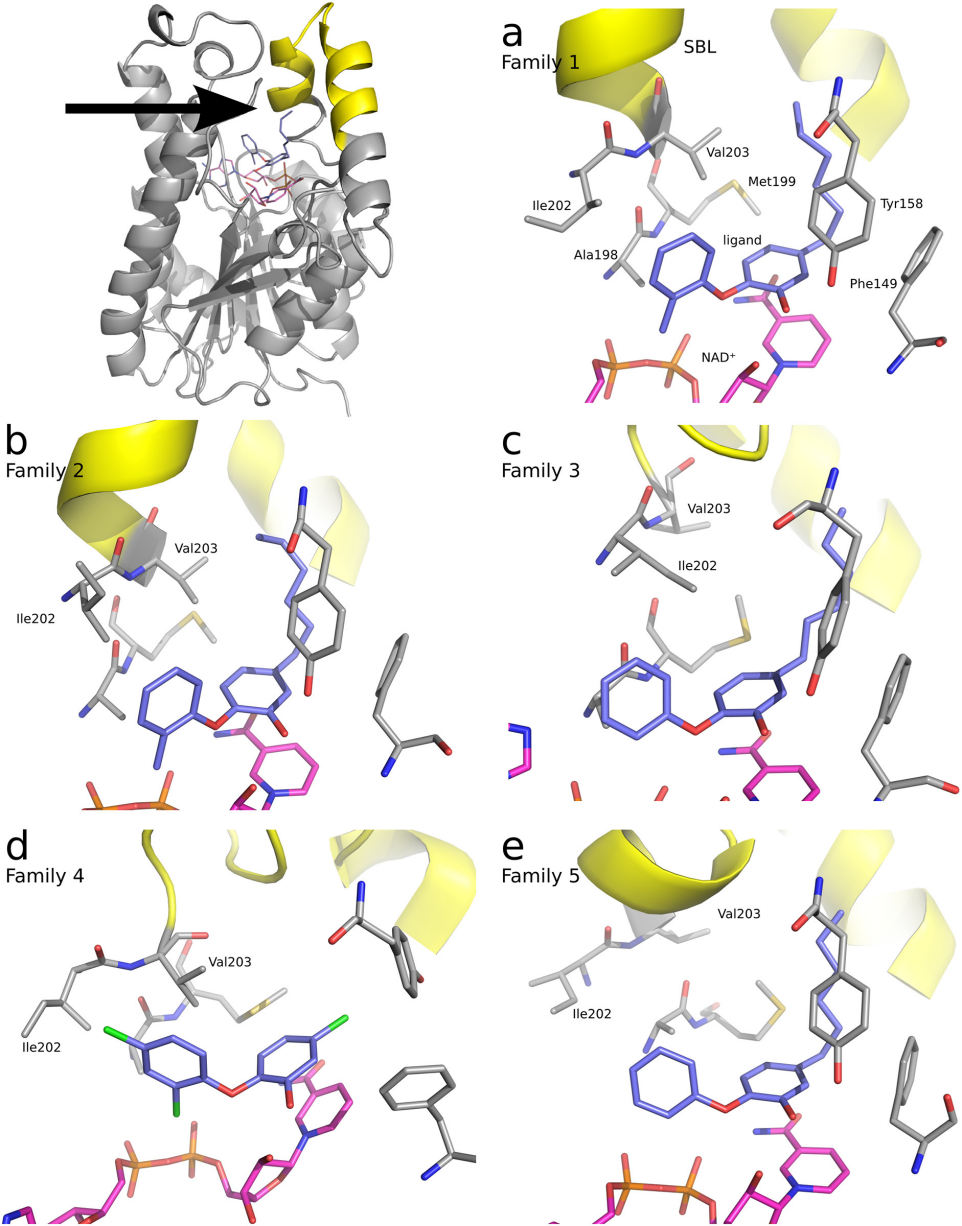
Illustration of conformational families of InhA. After summarizing the eight clusters of the hierarchical cluster analysis to five conformational families, a Partitioning Around Medoids (PAM) clustering was performed with R for each conformational family. The resulting medoids are illustrated as cluster representatives. The top left Fig Shows an entire monomer (A) of InhA from the crystal structure of the complex with **PT70** (PDB 2X23). The substrate binding loop (SBL) is highlighted in yellow. The arrow represents the direction of the view for the subsequent images. **(a)** Family 1: crystal structure conformation; **PT70** monomer 4 after 34 ns of MD simulation. SBL and pocket residues are labeled. The ligand carbon atoms are depicted in slate blue, the cofactor carbon atoms in magenta. **(b)** Family 2: Helical twist of ascending SBL branch with Ile202 shifted toward the ligand; **PT70** monomer 3 after 141 ns of MD simulation. **(c)** Family 3: Enhanced movement of Ile202 far into the hydrophobic cavity; **6PP** monomer 1 after 102 ns of MD simulation. **(d)** Family 4: Flip of Tyr158 toward the hydrophobic pocket; **TCL** monomer 3 after 119 ns of MD simulation. **(e)** Family 5: Ile202 movement toward the outside of the protein into the solvent; **6PP** monomer 3 after 27 ns of MD simulation.

The hierarchical cluster analysis was carried out with R [20] using the complete linkage method. This method was preferred over others not only because it tends to produce clusters with similar diameter, but primarily because it provides readily interpretable results in terms of a maximum RMSD value between members of a cluster. Here, eight clusters of recurring conformations of the InhA binding pocket were identified at an RMSD cutoff of 3.5 Å (cf. Supporting Information S2 Fig for further details). On the basis of the cluster dendrogram and the corresponding structural similarities, the clusters were further summarized to five “monophyletic” conformational families. Subsuming the clusters to monophyletic families was achieved by visual inspection instead of raising the RMSD cutoff, since mere RMSD values might overestimate the importance of minor backbone movements while concealing important side chain flips. These families are hereinafter referred to as Families 1 to 5 (cf. Fig 3):

- Family 1 (based on cluster 1) corresponds to the crystal structure conformation of the **PT70**-complex;
- Family 2 (based on clusters 2 and 3) shows a conformation with a slight twist of helix *α*6 (residues 202–209 in the ascending branch of the SBL), resulting in a shift of Ile202 toward the ligand and a minor displacement of Val203 toward the hydrophobic pocket;
- Family 3 (based on clusters 4 to 6) is characterized by a more open conformation of helix *α*6 and new positions of Ile202 and Val203: Ile202 now adopts the position of Val203 in the **PT70**-crystal structure, and Val203 is shifted to the back, farther away from the binding pocket;
- Family 4 (based on cluster 7) represents the conformations with a flip of Tyr158 toward the hydrophobic pocket and an associated conformational change of Phe149 toward the former position of Tyr158;
- Family 5 (based on cluster 8) is characterized by another open conformation of helix *α*6 resulting in a shift of Ile202 and Val203 toward the outside.

The quantitative analysis of the conformational families shows that Family 1 is by far the most frequent conformation, accounting for 58.21% of the frames across all monomers of the three simulations (cf. Table 1 and Supporting Information S3 Fig). Family 2 occurs with a frequency of 19.98%, whereas Family 3 accounts for 13.75%. Families 4 and 5 constitute the minority of conformations with 7.06% and 0.99%, respectively.

**Table 1:** Occurrence frequencies (in %) of the conformational families of the InhA binding pocket in the three analyzed simulations of the PT70-, 6PP- and TCL-complexes, based on the hierarchical clustering analysis.

	Family 1	Family 2	Family 3	Family 4	Family 5
*PT*70_1_	4.97	3.37	0.00	0.00	0.00
*PT*70_2_	8.33	0.00	0.00	0.00	0.00
*PT*70_3_	6.79	1.55	0.00	0.00	0.00
*PT*70_4_	7.89	0.44	0.00	0.00	0.00
6*PP*_1_	3.53	1.10	3.70	0.00	0.00
6*PP*_2_	5.30	0.00	3.04	0.00	0.00
6*PP*_3_	0.66	4.75	1.93	0.00	0.99
6*PP*_4_	5.85	2.48	0.00	0.00	0.00
*TCL*_1_	2.48	5.85	0.00	0.00	0.00
*TCL*_2_	8.33	0.00	0.00	0.00	0.00
*TCL*_3_	1.27	0.00	0.00	7.06	0.00
*TCL*_4_	2.81	0.44	5.08	0.00	0.00
*Sum*	58.21	19.98	13.75	7.06	0.99

Breaking this down to the individual simulations shows a clear difference between **PT70** and the other two complexes: Whereas Family 2 and 3 conformations show an occurrence of only 16.1% and 0.0%, respectively, in the simulation of the **PT70**-complex, values of 25.0% (Family 2) and 26.0% (Family 3) are obtained for the **6PP** simulation and values of 18.9% (Family 2) and 15.2% (Family 3) for the **TCL** simulation. Besides that, the **TCL** simulation shows 21.2% Family 4 conformations and the **6PP** simulation 3.0% Family 5 conformations. Thus, Family 1 conformations are found to 83.9% in the **PT70** simulation, but only to 46.0% in the **6PP** and to 44.7% in the **TCL** simulation (cf. Supporting Information S3 Fig).

Apparently, while the state corresponding to conformational Family 1 is stably maintained by the **PT70**-complex, the **6PP**- and **TCL**-complexes have a clear tendency to depart from this state (cf. part b of Supporting Information S2 Fig). Interestingly, this is not simply due to a reduced occupation of the hydrophobic pocket, because both **PT70** and **6PP** occupy this site with a hexyl chain, while **TCL** projects only a chlorine substituent into the pocket. However, the space left unoccupied by **TCL** is the reason why the Tyr158 side chain can switch its orientation and lead to a Family 4 conformation, which occurs only in the **TCL**-simulation and is not possible with the other complexes.

### SBL dynamics and secondary structure analysis

**Fig 4:**
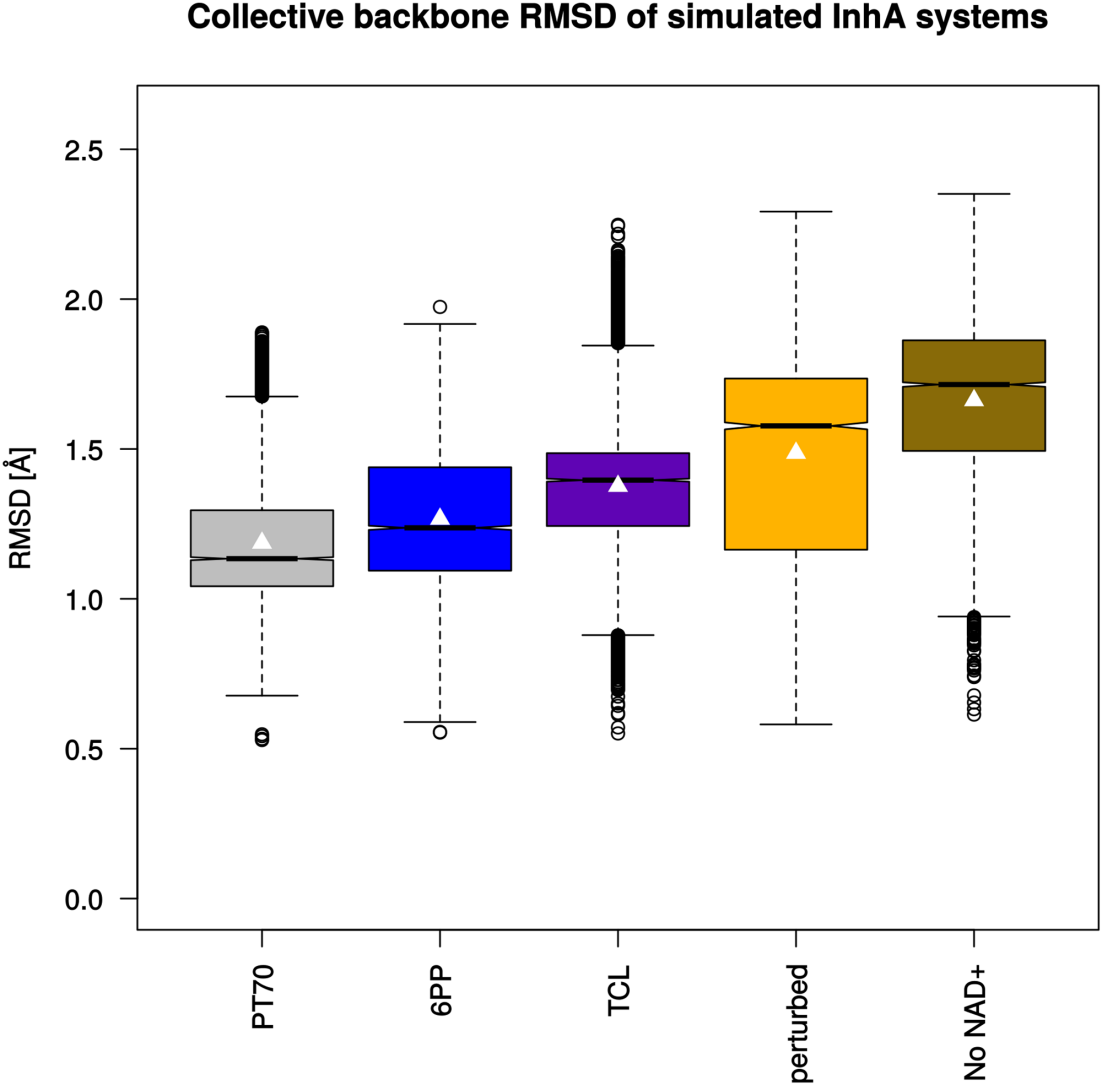
Collective backbone RMSD values (C, N, and C_*α*_ atoms) of InhA monomers. Each monomer of the simulated homotetrameric systems (150 ns) was fitted individually onto chain A of the 2X23 crystal structure as reference for the RMSD measurements and the data of the four monomers were combined to one box plot per system. Boxes indicate the interquartile range (first to third quartile), black lines in the boxes show the median of each distribution. The whiskers extend to values 1.5 times the interquartile range from the box. Significant differences in the medians are indicated by non-overlapping notches. Average values are marked by white triangles.

As the ordering of the SBL is supposed to play an important role in slow-binding inhibition of InhA [6, 7, 13], the dynamic behavior of this structural segment deserves special attention. To look first at the overall backbone dynamics of the entire systems, the RMS deviation of the backbone atoms of each monomer was calculated with respect to chain A of the 2X23 crystal structure (Fig 4). All ligand-bound systems show high stability of the overall structure throughout the entire simulation. With averages of 1.19 Å and 1.27 Å, the **PT70** and **6PP** complexes display slightly lower RMS deviations than the complex with **TCL** (1.38 Å). Not unexpectedly, the perturbed systems without ligand show a clear shift toward higher values and larger fluctuations. Nevertheless, the medians and averages remain well below 2 Å in all cases, indicating reasonable stability of the entire trajectories.

**Fig 5:**
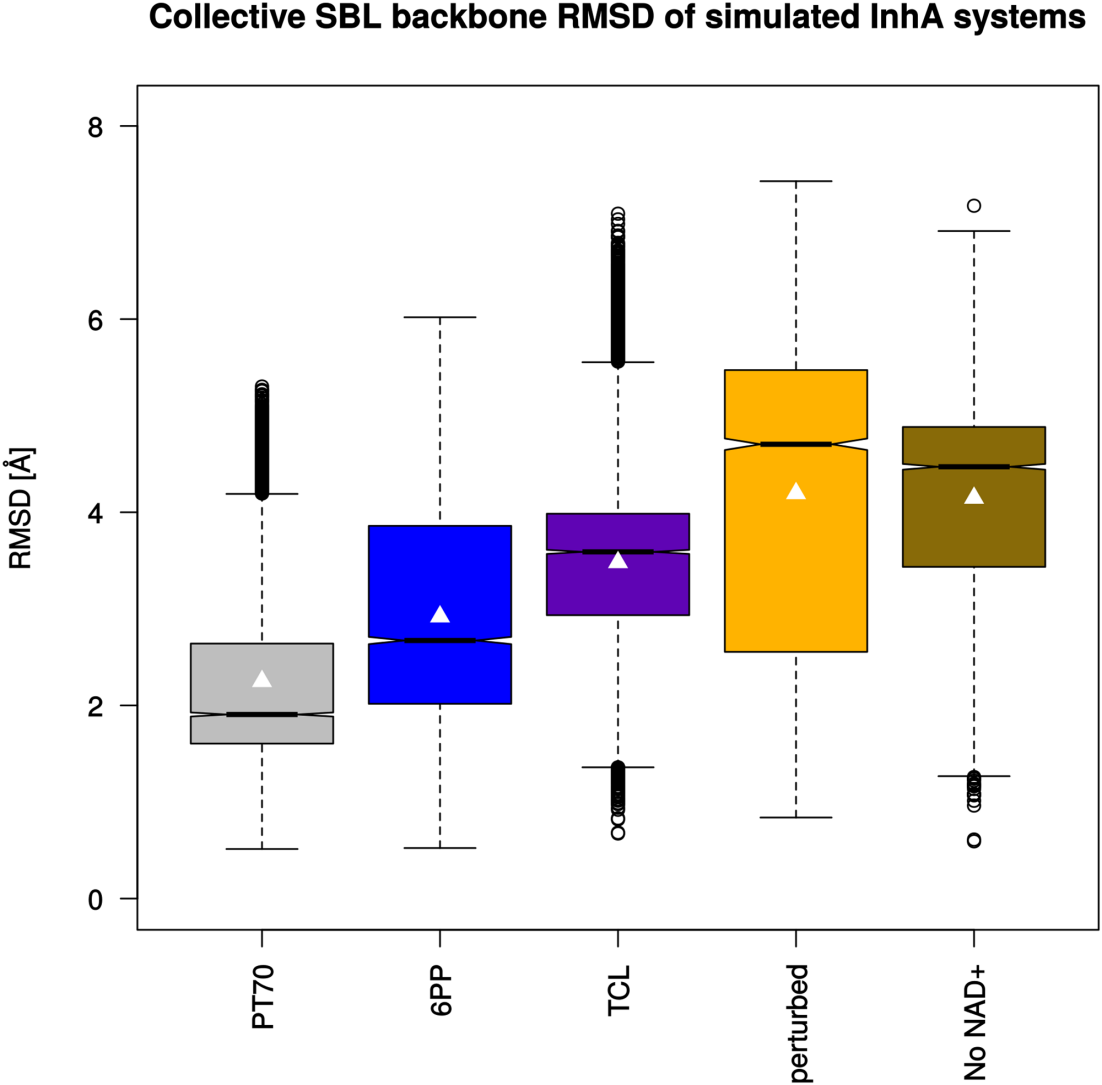
Collective backbone RMSD values of the substrate binding loop in the InhA monomers. Each monomer of the simulated homotetrameric systems (150 ns) was fitted individually onto chain A of the 2X23 crystal structure as reference for the RMSD measurements and the data of the four monomers were combined to one box plot per system. See Fig 4 for further explanations.

With these values as reference, the large degree of flexibility of the SBL becomes immediately evident. The RMSD of the backbone atoms between residues 202 and 218 (corresponding to the entire SBL) shows similar overall trends as seen in the analysis of the complete backbone, but (much) larger absolute values and fluctuations (Fig 5). In fact, the major mobility of the backbone is observed in the SBL. The highest RMSD values (as for example in the *perturbed* system) correspond to completely opened loop conformations. Thus, the time scale of the simulation is sufficient to encounter major loop disordering and opening. Furthermore, partial or complete loop closing and rearrangement can be seen after some opening events (e.g., **6PP** monomer 4; cf. Supporting Information S4 Fig, which shows the RMSD of the SBL as a function of time for each monomer of the simulated systems), emphasizing that the produced trajectories do not simply evolve toward a growing disorder.

**Fig 6:**
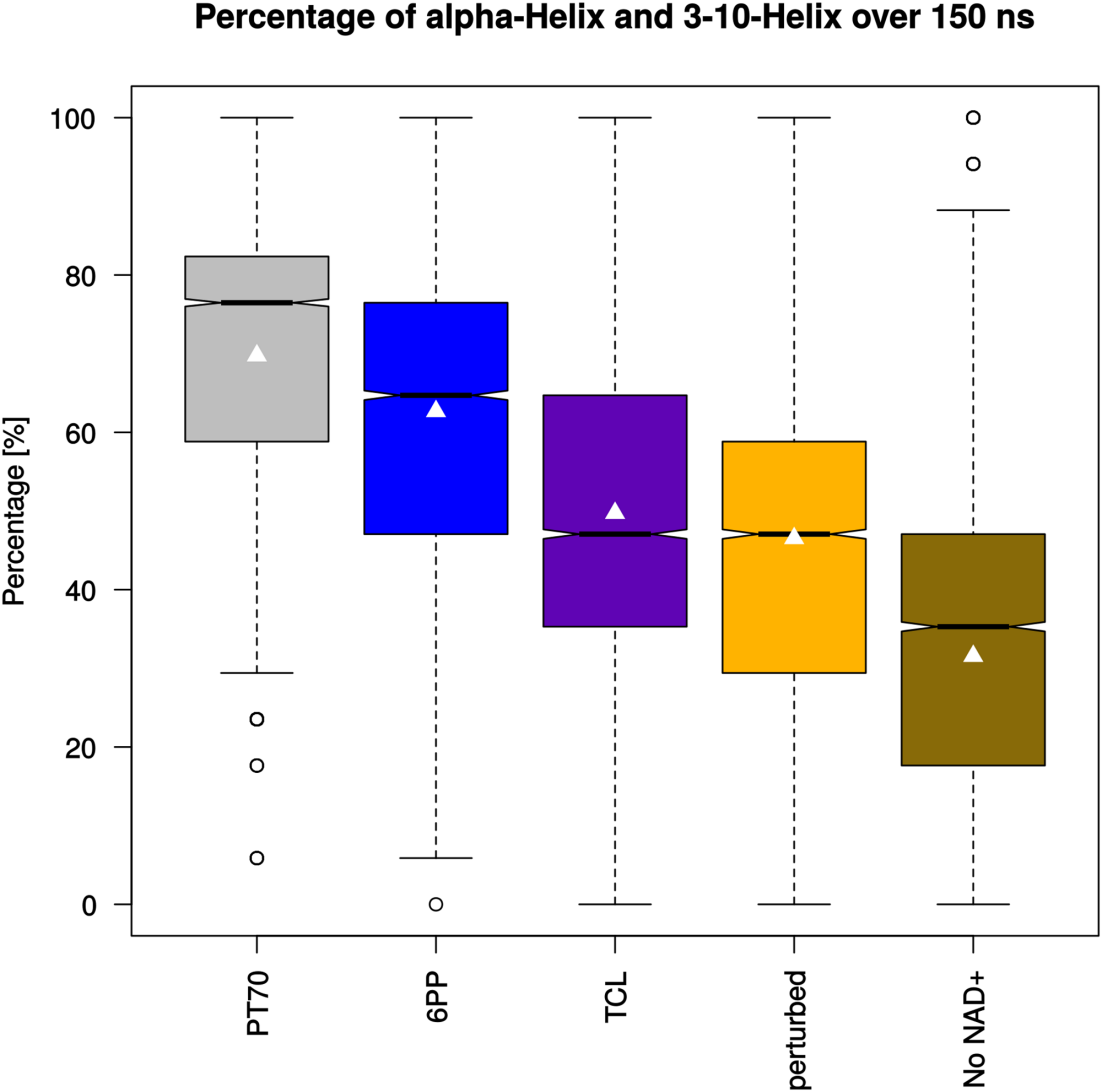
Occurrence frequency (in % of the trajectory snapshots) of *α*-helix and 3_10_-helix motifs in the substrate binding loop. Each monomer of the simulated homotetrameric systems (150 ns) was analyzed, and data of the four monomers were combined to one box plot per system.

Since the ordering of the two helical SBL branches is important for inhibitor binding and happens primarily at the secondary-structure level, a secondary-structure analysis was performed using the VMD plug-in *Timeline* to assign one of six secondary-structure motifs to each atom of the SBL backbone atoms (residues 202 to 218) for each frame of the trajectory: (1) isolated bridge, (2) Coil, (3) 3_10_-helix, (4) *α*-helix, (5) *π*-helix, and (6) turn [21]. The 2X23 crystal structure SBL consists completely (100%) of *α*-helix and 3_10_-helix atoms. For the simulations, the average percentage of these two motifs was calculated over the entire sampling time (Fig 6). With an average of 69.76% the **PT70**-bound monomers show the highest percentage of *α*-helix and 3_10_-helix motifs during the simulation, followed by **6PP** (62.67%). With 49.73% the **TCL** monomers are comparable to the *perturbed* monomers (46.52%). *No NAD^+^* shows by far the lowest percentage of these helical motifs (31.55%). This reinforces the notion that the proper occupation of the hydrophobic pocket is an important contributor to the conservation of the helical SBL structure of the final conformational state EI*. The lower helical-motif frequency of **6PP** and **TCL** compared to **PT70** is in line with their differences in binding affinity and residence time, stressing the importance of long-term SBL conservation.

### Hydrogen-bond interactions and binding mode analysis

**Fig 7:**
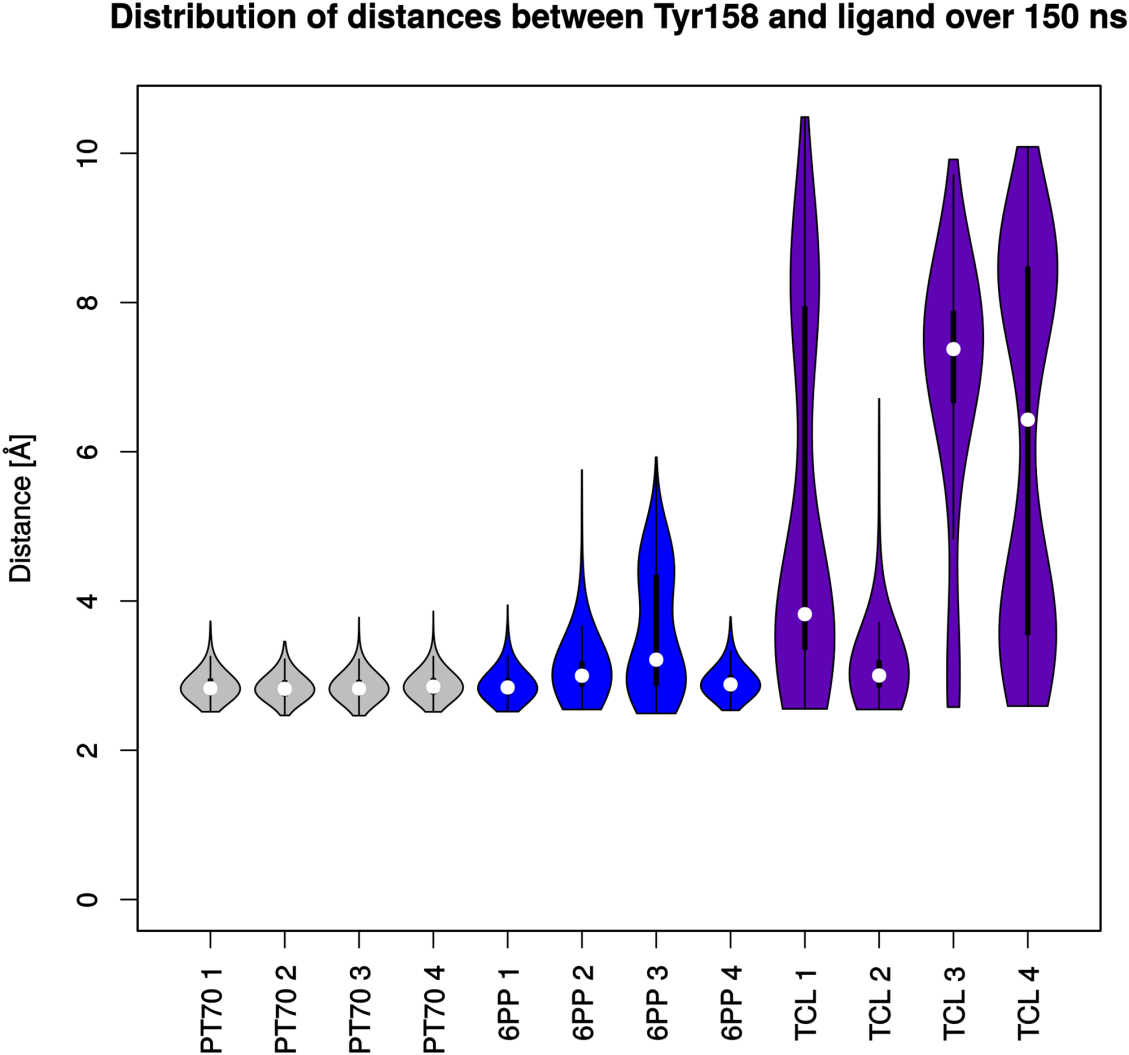
Violin plots of distances between the phenolic oxygen of Tyr158 and the respective ligands. White dots depict the medians. Thick vertical lines indicate the interquartile ranges (IQR), thin lines extend to 1.5 ⋅ IQR from the third and first quartile, respectively. The shape of the violins illustrates the kernel density estimation of the respective distribution.

We now focus on the ligand and analyze first the hydrogen bond between Tyr158 and the A-ring phenolic oxygen, which is a highly conserved interaction between diphenyl ethers and InhA. For analysis, the distance between the Tyr158 oxygen (OH) and the phenolic oxygen of the ligands was followed over the entire trajectory (cf. Fig 7). **PT70**-bound monomers show by far the lowest distance with medians ranging from 2.82 Å to 2.85 Å, followed by **6PP** (2.84 Å to 3.21 Å) and **TCL** (3.00 Å to 7.34 Å). The bimodal distributions observed for the **TCL** monomers 1 and 4 are caused by the transition to an alternative binding mode of **TCL** further described below. The shorter distances for **6PP** and especially **PT70** evince that the differences in the chemical structures of the ligands directly influence the formation and maintenance of the important hydrogen bond between Tyr158 and the ligands. The measured distances correlate with the relative affinity of the ligands (Fig 2), showing a stably maintained hydrogen bond for **PT70**, a partially maintained hydrogen bond for **6PP**, and a hardly stable interaction for **TCL**.

**Fig 8:**
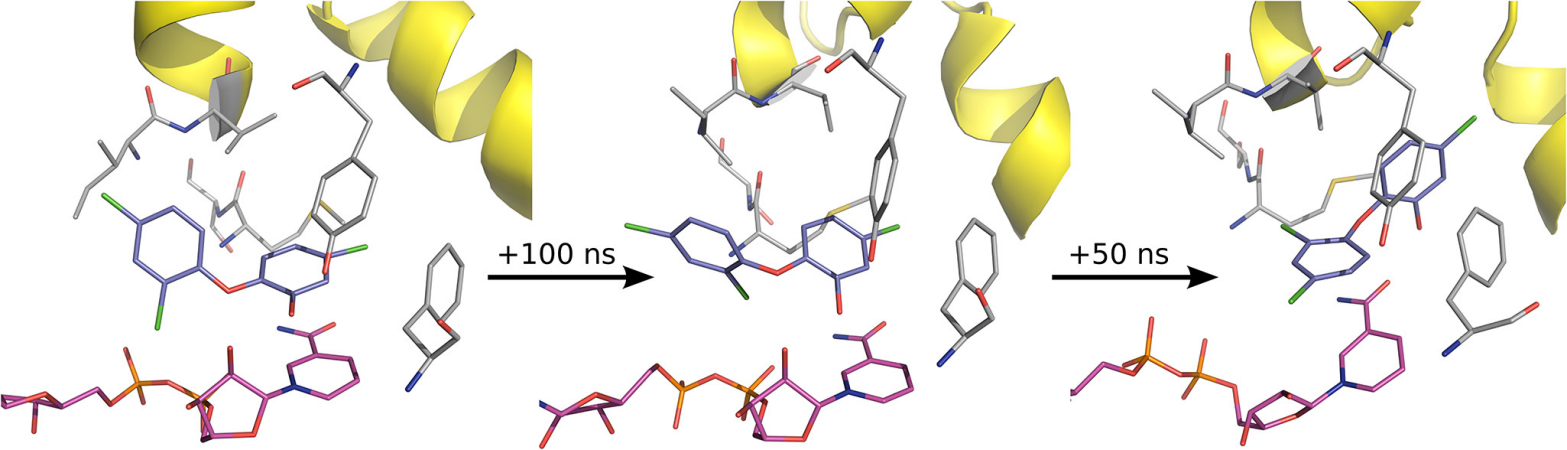
TCL of monomer 1 at 0 ns, 100 ns, and 150 ns of MD simulation, respectively. The initial change of binding mode can be observed at 100 ns, resulting in the final binding mode shortly after, which stays stable until the end of the simulation (150 ns). Very similar observations were made for **TCL** monomer 4, but starting already at 70 ns (cf. also Fig 9).

The second most important aspect of the diphenyl ether binding mode is the occupation of the hydrophobic pocket. While **PT70** and **6PP** both fill the pocket almost completely (a calculation of the free pocket volume with POVME [22] shows virtually no free volume for both complexes), the bound **TCL** leaves free space to be occupied. In fact, this space is flooded by water molecules after a few hundred picoseconds (cf. for example **TCL** monomer 2, Supporting Information S5 Fig). Although this may appear counterintuitive based on the lipophilic character of this area, it is well known that given sufficient space and accessibility, water molecules also occupy lipophilic binding sites [23]. The most drastic effect of the missing hydrophobic moiety of **TCL** can be observed in **TCL** monomers 1 and 4, where the ligand changes its binding mode entirely after around 100 ns and 70 ns, respectively (Fig 8).

**Fig 9:**
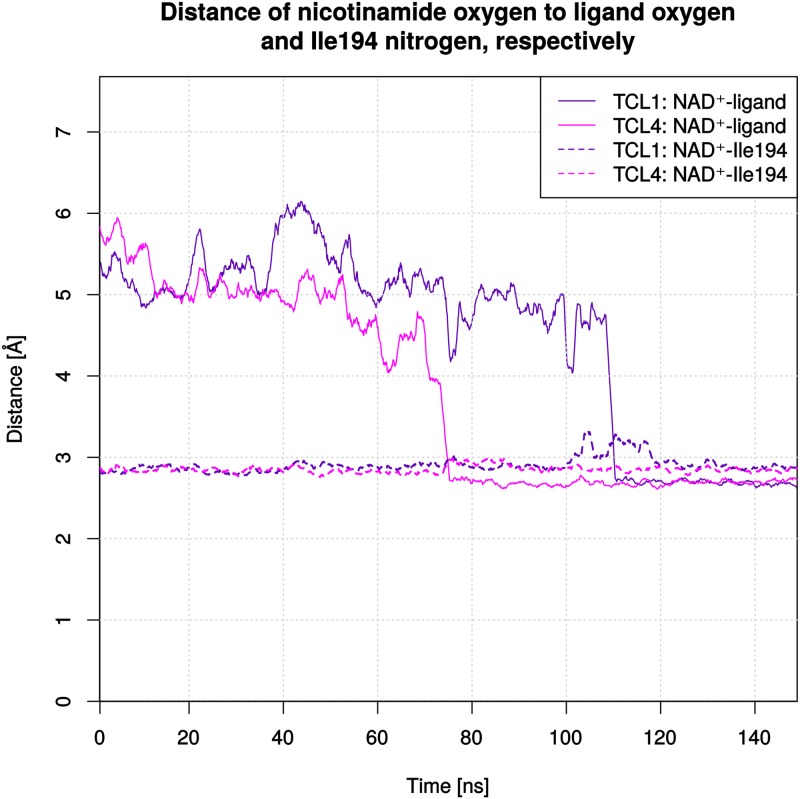
Distances between the NAD^+^ nicotinamide oxygen and the phenolic oxygen of the ligand or the Ile194 backbone nitrogen. Distances are shown as a function of time in a moving-average plot with a window of 20 frames. Monomers 1 and 4 are illustrated for the **TCL** complex. Continuous lines indicate distances to the ligand, whereas dotted lines are used for distances to Ile194. For each illustrated ligand a stable interaction with a distance below 3 Å can be observed after the binding-mode change, while the interaction of NAD^+^ with Ile194 (present in the starting structure) is only slightly affected.

The new binding mode displays a breaking of the hydrogen bond between Tyr158 and the phenolic oxygen of the diphenyl ether with subsequent shift into the hydrophobic pocket. After the scaffold transition, the A-ring, formerly stacked above the nicotinamide ring system, now occupies the hydrophobic pocket and forms a polar interaction with the nicotinamide oxygen. The B-ring is now placed at the former location of the A-ring. In both cases a stable interaction with the nicotinamide oxygen is observed once the binding mode has changed. This is also represented by heavy-atom distances below 3 Å (Fig 9). This new interaction could also be observed in MD simulations of the *Plasmodium falciparum* enoyl-ACP reductase (*Pf*ENR) in complex with NAD^+^ and the ligands **FT0** and **FT1**, respectively [24]. The novel binding mode of **TCL** co-occurs with the conformational Families 2 and 3, suggesting that a shifted Ile202 is detrimental to ligand stabilization in the pocket. There is indeed a steric hindrance between Ile202 and the B-ring chlorine of the ligand after Ile202 has moved. As a result, the ligand is pushed from “above” and eventually forced to rotate its B-ring, whereupon it yields and moves toward the hydrophobic pocket. Please note that this new binding mode is not postulated as an actual alternative binding mode of **TCL**. Rather, it is a consequence of the instability of the artificial starting structure and only shows that an alternative interaction with the cofactor might be possible in the binding pocket. Because this interaction requires a Family 2 or Family 3 conformation, it does, however, not provide a strategy to increase the residence time of slow-binding inhibitors.

### Influence of *ortho*-substituted B-ring

**Fig 10:**
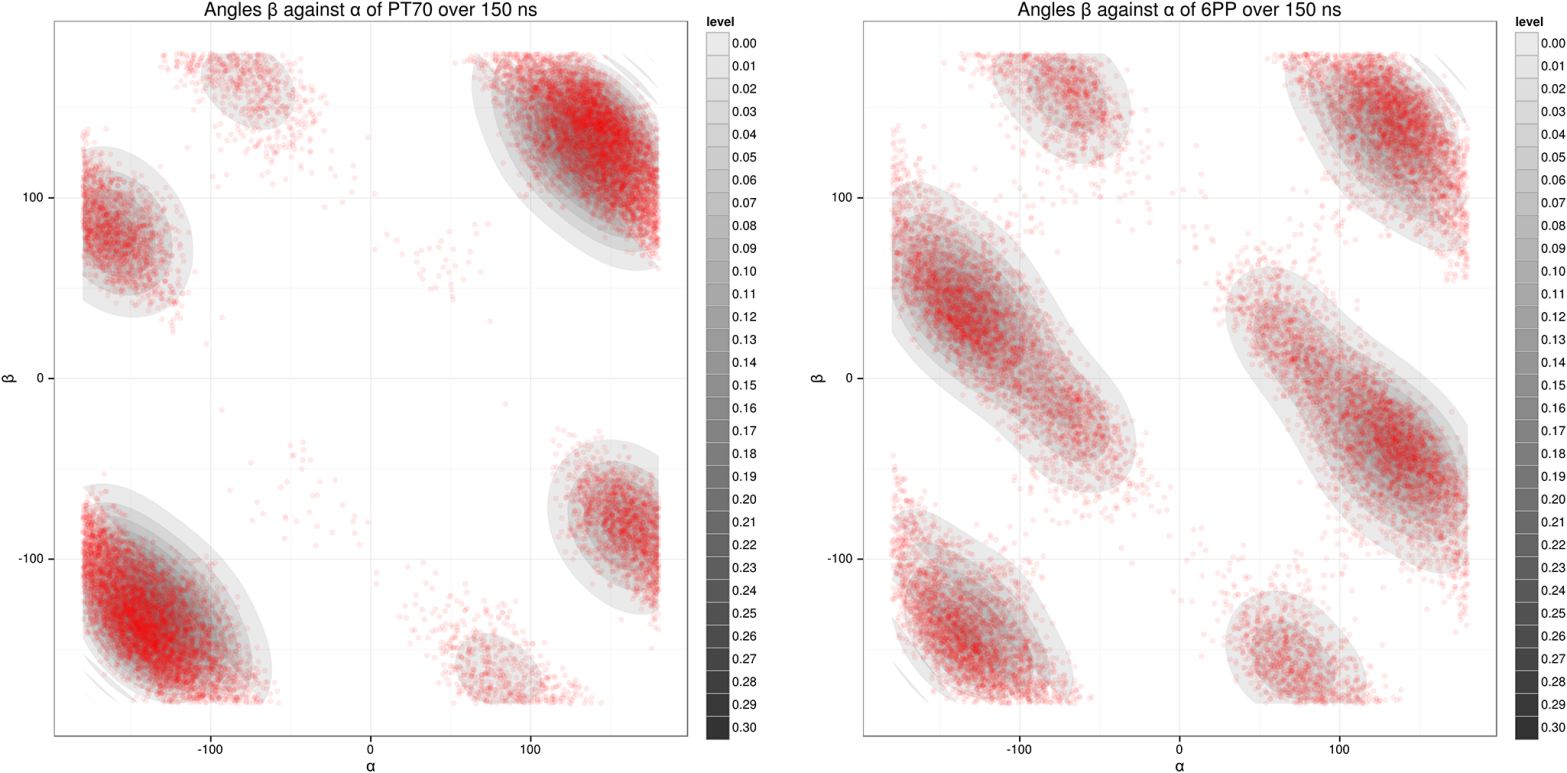
2D density plot for the ether dihedral angles *α* and *β* of the unbound ligands PT70 (left) and 6PP (right) based on a 150 ns MD simulation in aqueous solution. The dihedral angles *α* (C_*OH*_-C-O-C) and *β* (C-O-C-C_*Me*/*H*_) are illustrated in Fig 2.

While **6PP** is a rapid reversible inhibitor, **PT70** binds with a residence time of 24 minutes (Fig 2), although the *ortho*-methyl group at the B-ring of **PT70** is the only structural difference [7, 13]. Interestingly, for the **6PP** complex the simulations indicate a reduced stability of the Family 1 state in comparison to the **PT70** complex, and the conformational Families 2 and 3 are significantly more frequent in the **6PP** simulation. With the *ortho*-methyl moiety as the only substitution, this difference appears as the logical origin for these observations. To investigate the effect of *ortho*-methyl substitution on the ligand conformations (which are mainly determined by the torsions around the two ether bonds), two additional 150 ns MD simulations were conducted for each ligand solvated in a water box. By measuring the dihedral angles of the ether moiety along the trajectory, a 2D density map of the (C-O-C-C_*Me*/*H*_)-dihedral *β* *versus* the (C_*OH*_-C-O-C)-dihedral *α* was generated for each ligand (Fig 10). The strong peaks in the distribution of the **PT70** angle pairs suggest that fewer conformations are populated compared to **6PP**. Hence, as expected, the *ortho*-substituted **PT70** is more constrained in its intramolecular mobility, hindering the Ile202 movement toward the hydrophobic pocket to a greater extent than the unsubstituted **6PP**. This very likely accounts for the enhanced occurrence of Families 2 and 3 in the case of **6PP** and for the (on average) larger RMS deviations and fluctuations of the ligand in the binding pocket (Table 2). Interestingly, also the hexyl chain of **6PP** shows higher mobility than the **PT70** hexyl chain in the binding pocket (Table 2, Supporting Information S6 Fig). In summary, the conformational stabilization of **PT70** by the *ortho*-methyl group appears to translate directly to increased SBL stabilization and retention of a Family 1 conformation.

**Table 2:** Trajectory averages and standard deviations of heavy-atom RMSDs of the PT70 and 6PP ligands and their hexyl chains, respectively. RMSDs were measured individually for each ligand in the four monomers with respect to the corresponding starting structure (after the heating cycles).

	all heavy atoms		hexyl chain	
	Avg. RMSD [Å]	SD [Å]	Avg. RMSD [Å]	SD [Å]
*PT*70_1_	1.15	0.19	1.50	0.50
*PT*70_2_	1.15	0.21	1.15	0.22
*PT*70_3_	1.14	0.18	1.20	0.27
*PT*70_4_	1.09	0.22	1.17	0.26
6*PP*_1_	1.24	0.23	1.77	0.48
6*PP*_2_	1.18	0.29	1.31	0.32
6*PP*_3_	1.32	0.28	1.91	0.49
6*PP*_4_	1.72	0.31	1.41	0.39

### Comparison with experimental structures

To further judge the relevance of the simulation results and to discuss the conformational families in the context of the EI and EI* states of the two-step binding process of slow-binding InhA inhibitors (Fig 1), a comparison with the experimentally available structural information is important. Most relevant in this context are the very recently released crystal structures of the ternary diarylether complexes with InhA and NAD^+^ from the studies of Li et al. [17] and Pan et al. [16]. These complexes with the slow-binding inhibitors **PT10** (PDB 4OXY), **PT91** (PDB 4OYR), **PT92** (PDB 4OHU) and **PT119** (PDB 4OIM) and the rapid-reversible inhibitor **PT155** (PDB 4OXN and 4OXK) show differences in the conformations of Ile202/Val203 and the orientation of helix *α*6 of the SBL. The complexes with **PT10**, **PT91** and **PT92** predominantly show the same binding-site conformation and helix orientation as the **PT70**-complex structure, strongly supporting the assumption that this corresponds to the EI* state (the ligands **PT10**, **PT91** and **PT92** differ from **PT70** only by a 2’-nitro, 2’-chloro and 2’-bromo substituent, respectively, instead of the 2’-methyl group) [17]. In contrast, the complex with **PT119** (carrying a 2’-cyano group) displays an alternative arrangement of Ile202 (which adopts the typical position of Val203) and Val203 (which is displaced to the back), but a relatively closed orientation of the helix [16]. Finally, the structures with the rapid-reversible 4-pyridone inhibitor **PT155** (carrying a 4-pyridone as A-ring and an additional 4’-amino substituent on the B-ring in comparison to **PT70**) not only show an unresolved SBL in the monomers of the asymmetric unit, but—for the first time—for one of the monomers also a fully resolved SBL with a widely open orientation of helix *α*6, which has been interpreted as a representation of the EI state by Li et al. [17].

**Fig 11:**
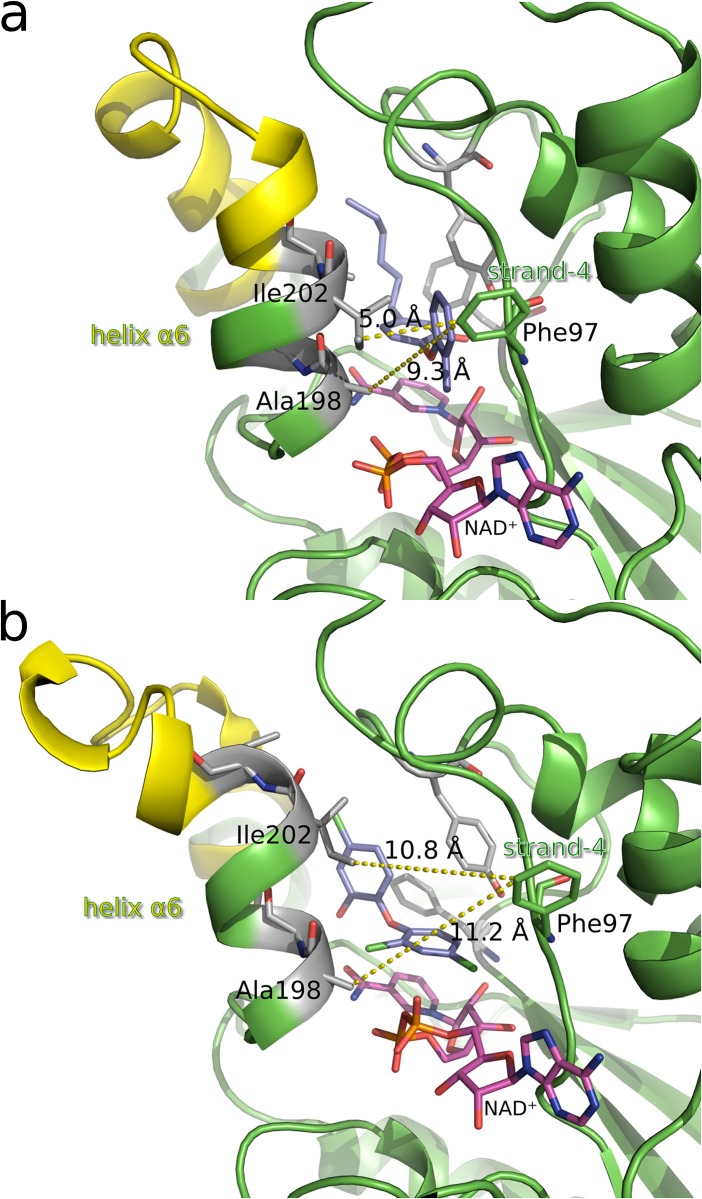
Open and closed conformations of InhA observed in the MD simulations. Figure (a) shows the closed state represented by the medoid of conformational Family 1, figure (b) illustrates the open state represented by the medoid of cluster 4 (belonging to conformational Family 3). The same view of the binding pocket as in Fig 3 of Li et al. [17] is used for better comparison. In this view, the portal-forming elements are located left (helix *α*6) and right (strand-4) of the binding site. The distances highlighted as yellow dashed lines were measured between Ala198/Ile202 on helix *α*6 and Phe97 on strand-4. For comparison, in the crystal structure of the **PT70** complex (PDB 2X23) respresenting the closed state, a distance of 4 Å is found between Ile202 and Phe97, whereas the open state is characterized by a distance of about 10 Å between Ala198 and Phe97 in chain B of the **PT155**-complex crystal structure (PDB 4OXN) [17].

A comparison of this **PT155**-structure with the conformational families suggests that Family 3 indeed captures the characteristics of the EI state: Ile202 is positioned above the ligand, Val203 is moved to the back, and helix *α*6 adopts a very open conformation. Fig 11b highlights this open state for a Family 3 representative: it shows a distance between helix *α*6 and strand-4 (used by Li et al. [17] to measure the degree of opening) of 11 Å, whereas only 5 Å are measured for Family 1 (Fig 11).

The complex with the slow-binding inhibitor **PT119** constitutes a special case, as it does not show the typical EI* conformation. Whereas in the EI* state Val203 in helix *α*6 is positioned closer to the ligand than Ile215 (located in helix *α*7), in the **PT119** structure Val203 is located far behind and Ile215 is close to the ligand [16]. Together with the altered position of Ile202, this conformation rather reminds of an EI-like state, albeit with a not fully open helix *α*6. Although the authors speculate about the relevance of this structure, they also note that “owing to the crystallization conditions and the potential impact from crystal packing, the observed structure for the **PT119** complex could represent a snapshot along the binding coordinate from EI to EI*” [16]. Indeed, very different crystallization conditions were used for this structure in comparison to the others, limiting the comparability. In particular, the very high acetate concentration leads to the observation of two acetate ions at potentially critical positions of the structure, namely between helix *α*6 and strand-4, as well as between helix *α*6 and helix *α*7. Accordingly, we assume that the EI*-state could not be reached under these conditions and that the conformation was frozen in an intermediate, but rather EI-like state. In our simulations, the particular conformational feature of **PT119** occurs only occasionally and only in the context of Family 3 conformations, supporting the EI-likeness (cf. Supporting Information S7 Fig, which illustrates the distance between Ile202 and Ile215 as a measure for the adoption of a **PT119**-like conformation).

In summary, the comparison with these newly released structures supports the notion that Family 1 corresponds to the EI*-state, whereas Family 3 may be considered as EI state. This has important implications for the interpretation of the simulations and the effects exerted by the different ligands.

### Determinants of residence time and implications for drug design

To optimize potential inhibitors regarding their residence time, it is desirable to understand the reasons which drive the conservation of an EI* state over time [5, 6]. Associating Family 1 conformations with the EI* macrostate and Family 3 conformations with the EI macrostate provides the possibility to interpret the simulation results in this context. Li et al. have carried out partial nudged elastic band MD simulations to investigate the free energy profile for the transition between EI and EI*, illustrating that the energy required for the arrangement of Ile202 and Val203 around the B-ring contributes directly to the height of the energy barrier for the transition from the EI to the EI* state [17]. In contrast, our classical MD simulations were all setup from the EI* state without a biasing potential, but introducing rapid-reversible inhibitors as perturbation to investigate their effects on the stability of the EI* state. As analyzed above, the simulations indeed show a clear tendency for major conformational changes (involving in particular Ile202 and Val203) toward an EI state in case of the rapid-reversible inhibitors **6PP** and **TCL** and a much higher stability in case of the slow-binding inhibitor **PT70**. Thus, the dynamic features revealed from the trajectories for the different systems may be linked to the substitution patterns of the examined diphenyl ethers to provide insights for rational ligand optimization toward longer residence times for InhA.

First of all, the *ortho*-methyl group of the B-ring has shown itself to be advantageous as an anchor. A substituent in this position occupies further space between helix *α*6 and cofactor. Moreover, it restrains the phenyl-oxygen-phenyl torsions (as shown in the simulations of the solvated ligands, cf. Fig 10), which stabilizes the ligand scaffold and thereby also the *para*-hexyl chain of the A-ring. This appears to improve the stable occupation of the hydrophobic pocket. Proper filling of the hydrophobic pocket is, in fact, a second major determinant, as evidenced by the **TCL**-simulation. In order to lock the binding pocket and the SBL in the EI* state (Family 1), it is desirable to prevent Ile202 and Val203 from moving toward helix *α*7 (residues 210 to 218). Thus, as a third factor and as a suggestion for ligand design, it could be beneficial to introduce a barricade group in 5’-position of the B-ring which might embed itself between Ile202 and Val203 and, thus, further stabilize them, possibly blocking Ile202 from traveling toward the hydrophobic pocket (cf. Fig 2). Thereby, the energy barrier between the EI* and the EI state might be significantly increased and the EI* complex could be maintained for a longer time. Notably, a substituent in this particular position is confined in its size by the adjacent Met103. Four *meta*-substituted ligands (fluoro, chloro, methyl and methoxy) were docked exemplarily into the InhA chain A binding pocket using Glide (version 5.8, Schrödinger, LLC, New York, NY, 2012) [25, 26] in extra precision mode (Supporting Information S8 Fig). All ligands show essentially the same binding mode as **PT70** in the crystal structure, underlining the availability of sufficient space for a small 5’-substituent of diphenyl ethers. Thus, new 2’-substituted diphenyl ethers with an additional small substituent in 5’-position are suggested as inhibitors with potentially further increased residence times.

## Conclusion

By using MD simulations with accumulated sampling in the low microsecond time scale, it was possible to unveil previously undetected conformational features of the *Mycobacterium tuberculosis* enoyl-ACP reductase InhA. Starting from an EI* state, the presence of rapid-reversible inhibitors caused an increased tendency for transitions to an EI-like state. The associated conformational changes and dynamic fluctuations of the protein binding pocket and the SBL were illustrated by the simulations. Analyses of conformations, pocket volume and secondary structure show different strategies for achieving structural conservation of the EI*-state over time and, thus, increased residence times of inhibitors: firstly, the occupation of the hydrophobic pocket and stabilization of Ile202 and Val203 to prevent these residues from turning over the hydrophobic pocket; secondly, the introduction of a barricade substituent in 5’-position of the B-ring to increase the energy required to arrange helix *α*6 around the B-ring, thus fashioning the final EI* state of **PT70**-like binding modes; and thirdly, the introduction of an anchor in *ortho*-position of the B-ring (methyl in **PT70**) to reduce the degrees of freedom with respect to the central diphenyl ether torsions. This limits the mobility of the bound ligand and, concomitantly, of the hydrophobic pocket, leading to lower fluctuations and an increased stability. These structural features not only keep the InhA binding pocket in the EI* state, but also directly influence the quality of the important hydrogen bond between Tyr158 and the ligand. Taken together, these findings provide valuable insights for future studies of inhibitor design directed against InhA.

## Methods

### Protein and ligand preparation

The highly ordered tetrameric InhA crystal structure with bound **PT70** and NAD^+^ (PDB code 2X23) [7] was used as starting point for the setup of all five simulation systems. Due to the high flexibility of the substrate binding loop the **TCL**-complexed crystal structure of InhA (PDB code 2B35) is incomplete in this crucial area. Therefore, a structural alignment of 2X23 and 2B35 was performed in PyMOL [27]. **TCL** was extracted from the 2B35 structure and placed into the ligand-free 2X23 protein, generating an uninterrupted InhA-NAD^+^-**TCL** complex. The ligand **6PP** was sketched and docked with Glide (version 5.8, Schrödinger, LLC, New York, NY, 2012) [25, 26] into the 2X23 crystal structure using standard precision. For each monomer the pose with the least RMSD from the crystallized **PT70** was chosen (0.58 Å, 0.47 Å, 1.03 Å, and 0.53 Å, respectively; calculated with fconv [28]). No crystal structure is available for the **6PP** complex, but comparison with the complex structures of the closely related ligands **5PP** and **8PP** [13] shows low RMS deviations (of 0.6 Å to 1.0 Å) between the **6PP** binding modes generated by docking and these ligands.

Hydrogen atoms were added to **PT70**, **TCL**, and **NAD^+^** with SYBYL-X. The Amber10 [29] module tleap was used for assigning the parameters of the ff99SB force field. RESP charges [30] were calculated for all three ligands and the cofactor based on HF/6-31G* electrostatic potentials obtained with Gaussian 03 [31]. With the Amber10 module parmchk [32] unavailable force field parameters were calculated according to the General Amber Force Field (GAFF) [33]. Atom and bond types of the ligand were assigned by antechamber [32].

### Molecular dynamics simulations

A short energy minimization of 200 cycles was performed using a generalized Born implicit solvent model [34, 35] as implemented in the Amber11 module sander [36]. Subsequently, the molecules were solvated with tleap using a TIP3P water box [37], retaining all crystallographic water molecules and adding sodium ions to ensure electro-neutrality. The resulting systems had dimensions of approximately 110 Å ⋅ 112 Å ⋅ 89 Å and contained about 101,000 atoms each. For heating-up, water molecules were allowed to move freely in the constant-volume box, while the proteins and ligands were kept rigid for 25 ps. During this step the systems were heated from 100 to 300 K for 20 ps and then cooled to 100 K over 5 ps by means of the Berendsen weak coupling algorithm [38] with a time constant of 0.5 ps. Then the complete systems were treated without constraints and gradually heated to 300 K over a time period of 25 ps. For each system a simulation of 150 ns at 300 K was then carried out, whereby covalent bonds to hydrogen atoms were constrained by the SHAKE algorithm and a time step of 2 fs was used. These simulations were run with NAMD 2.9 [39, 40] using the assigned force field parameters. Energetical equilibration of the simulation box was observed within 1.5 to 3 ns in all cases. The systems were treated with periodic boundary conditions. A van-der-Waals interaction cutoff of 12 Å was used, as well as the particle mesh Ewald methodology (PME) for electrostatic interactions [41]. Constant pressure was assured by the Nosé-Hoover Langevin piston pressure control [42, 43], while constant temperature was achieved by the use of Langevin dynamics. Additionally, two simulations of the uncomplexed ligands **PT70** and **6PP**, respectively, were conducted for 150 ns. Trajectory snapshots were saved every picosecond. For visual and statistical analyses, trajectory snapshots at intervals of 100 ps were considered, resulting in 1500 frames per system. The diphenyl torsion analyses (ligand-only simulations) were carried out with snapshots at 10 ps steps (i.e., 15000 data points). All analyses were performed with VMD and associated plug-ins [44]. All trajectories of the individual monomers were fitted to the chain A backbone atoms (C, N, and C_*α*_) of the 2X23 crystal structure with the *RMSD Trajectory Tool* of VMD for visual inspection and all quantitative analyses. Statistical analysis and plotting was done with the statistical framework R [20, 45, 46]. The pocket volume analysis was performed with POVME [22]. Structural visualizations were created with PyMOL [27].

## Supporting Information

S1 Fig 12x12 2D RMSD plot of the binding site (defined by the heavy atoms of Phe149, Tyr158, Ala198, Met199, Ile202, and Val203) of all PT70, TCL and 6PP monomers. RMSD values between two frames are illustrated according to the color scale on the right. The axes correspond to the simulation time (0 to 150 ns for each monomer). A single small box (square delimited by thin black lines) represents the comparison of the trajectory snapshots either within a given monomer (boxes along the diagonal) or between two different monomers (off-diagonal boxes). The bold black lines enclose the monomers of a particular homotetramer (i.e., **PT70**, **TCL** or **6PP**).
(TIF)

S2 Fig Hierarchical clustering analysis of binding-pocket conformers of the PT70, TCL and 6PP simulations based on the mutual RMSD comparison of the individual snapshots as shown in the 2D RMSD plot (Supporting Information S1 Fig). The calculated RMSD is used as distance measure with complete linkage. The clusters detected at an RMSD cutoff of 3.5 Å are shown in different colors and are numbered as explained in the text. **(a)** Cluster dendrogram. **(b)** Time line of cluster membership. For each monomer of the simulated systems all snapshots included in the analysis from 0 to 150 ns (at intervals of 1 ns) are consecutively written in a line as blocks of 30 ns. The numbers represent the cluster to which a particular snapshot belongs to. Family membership is highlighted by colors according to the legend at the bottom.
(TIF)

S3 Fig Cumulative frequencies of conformational families of the InhA binding pocket in 150 ns of the PT70, 6PP, and TCL MD simulations. Horizontal lines separate the single monomers of each of the three considered homotetrameric complexes.
(TIF)

S4 Fig Backbone RMSD plots of InhA SBL (residues 202 to 218) of single monomers. A moving average with a window size of 20 frames was used. The RMSD was measured with reference to chain A of the 2X23 crystal structure.
(TIF)

S5 Fig Snapshots of TCL monomer 2 after heating (0 ns, left) and after 700 ps of MD simulation (right). The ligand **TCL** is depicted in slate blue, the cofactor in magenta and the pocket residues including Leu218 in gray. The SBL is shown in yellow. Ligand, cofactor, and pocket residues are also shown as surface (wheat), oxygens of water molecules are shown in red. Flooding of the hydrophobic pocket is noticeable after 700 ps (right).
(TIF)

S6 Fig Heavy-atom RMSD distributions of hexyl chains of PT70 and 6PP. As references the respective coordinates of the starting structure (after the heating cycles) were used (cf. Fig 4 for further explanations).
(TIF)

S7 Fig Distance between the C_*β*_-atoms of Ile202 and Ile215 over time. **6PP** monomers 1 and 3 are shown in shades of blue, **TCL** monomer 4 is depicted in purple. The green baseline illustrates the **PT119** crystal structure (PDB 4OIM).
(TIF)

S8 Fig Top-ranked docking poses of four 5’-substituted PT70-like diphenyl ethers. Met103 is illustrated in green, the docked ligands are shown in salmon. **PT70** (shown in slate blue as reference) was substituted in 5’-position with a **(a)** fluoro-, **(b)** chloro-, **(c)** methyl-, and **(d)** methoxy-substituent. Docking was carried out with Glide in XP-mode using default settings and a maximum output of 10 poses per ligand.
(TIF)
