# Supplementary figures and images for: Slow-Onset Inhibition of Mycobacterium tuberculosis InhA: Revealing Molecular Determinants of Residence Time by MD Simulations
Source: PLoS One. 2015 May 21;10(5):e0127009. doi: 10.1371/journal.pone.0127009 (PMC4440617; doi:10.1371/journal.pone.0127009)

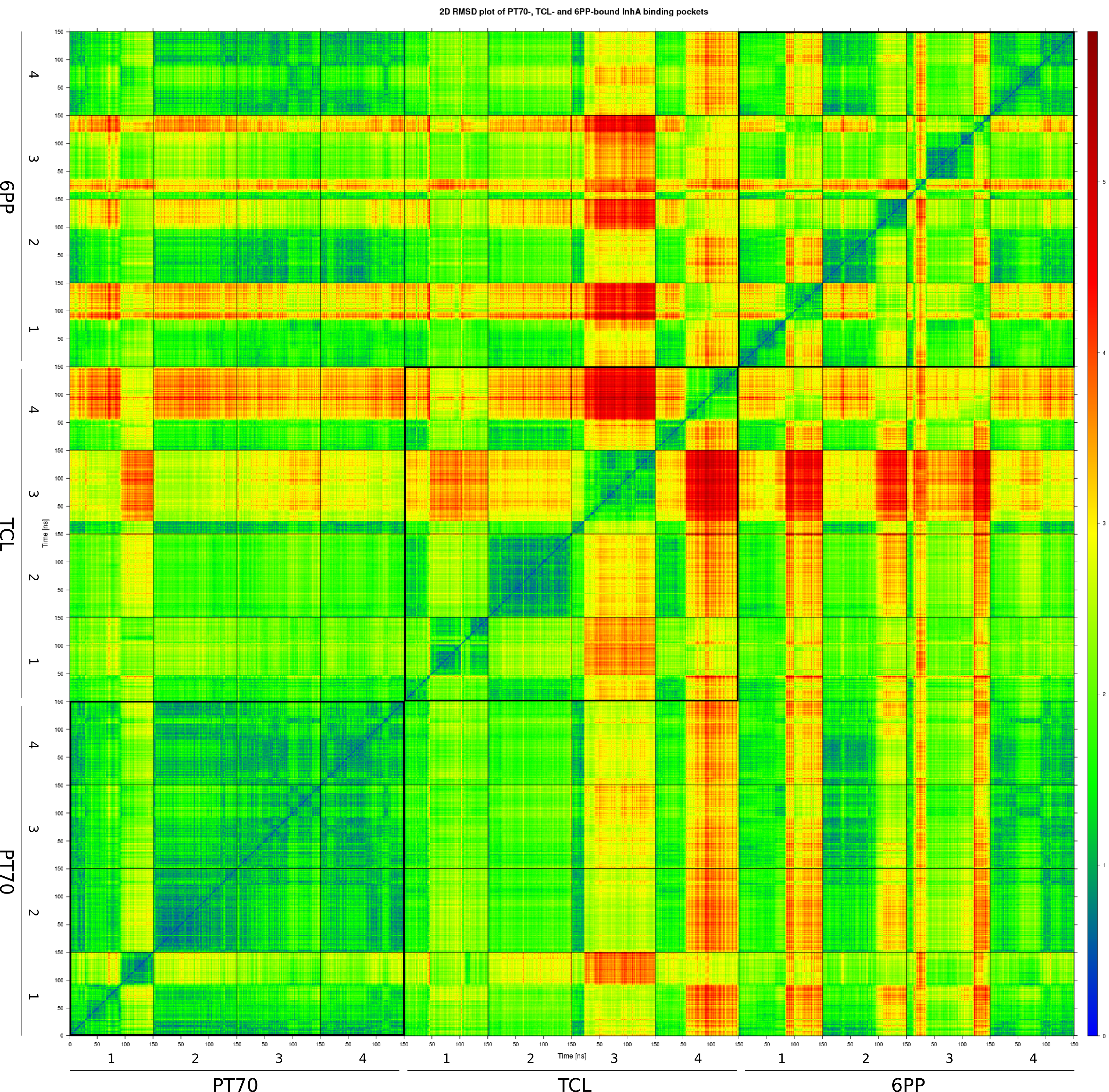

Supplement: S1 Fig — RMSD values between two frames are illustrated according to the color scale on the right. The axes correspond to the simulation time (0 to 150 ns for each monomer). A single small box (square delimited by thin black lines) represents the comparison of the trajectory snapshots either within a given monomer (boxes along the diagonal) or between two different monomers (off-diagonal boxes). The bold black lines enclose the monomers of a particular homotetramer (i.e., PT70, TCL or 6PP). (TIF) [file pone.0127009.s001.tif]

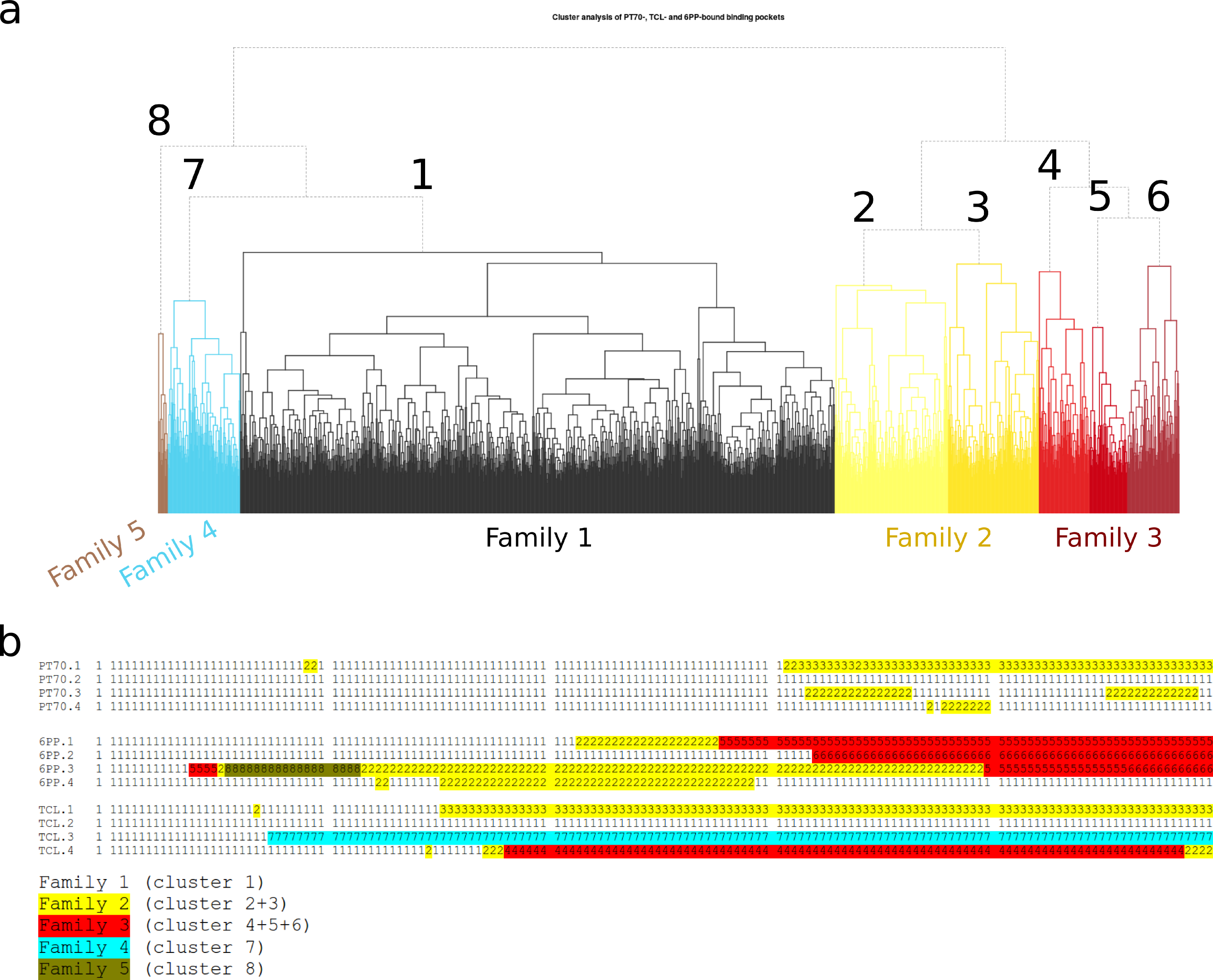

Supplement: S2 Fig — The calculated RMSD is used as distance measure with complete linkage. The clusters detected at an RMSD cutoff of 3.5 Å are shown in different colors and are numbered as explained in the text. (a) Cluster dendrogram. (b) Time line of cluster membership. For each monomer of the simulated systems all snapshots included in the analysis from 0 to 150 ns (at intervals of 1 ns) are consecutively written in a line as blocks of 30 ns. The numbers represent the cluster to which a particular snapshot belongs to. Family membership is highlighted by colors according to the legend at the bottom. (TIF) [file pone.0127009.s002.tif]

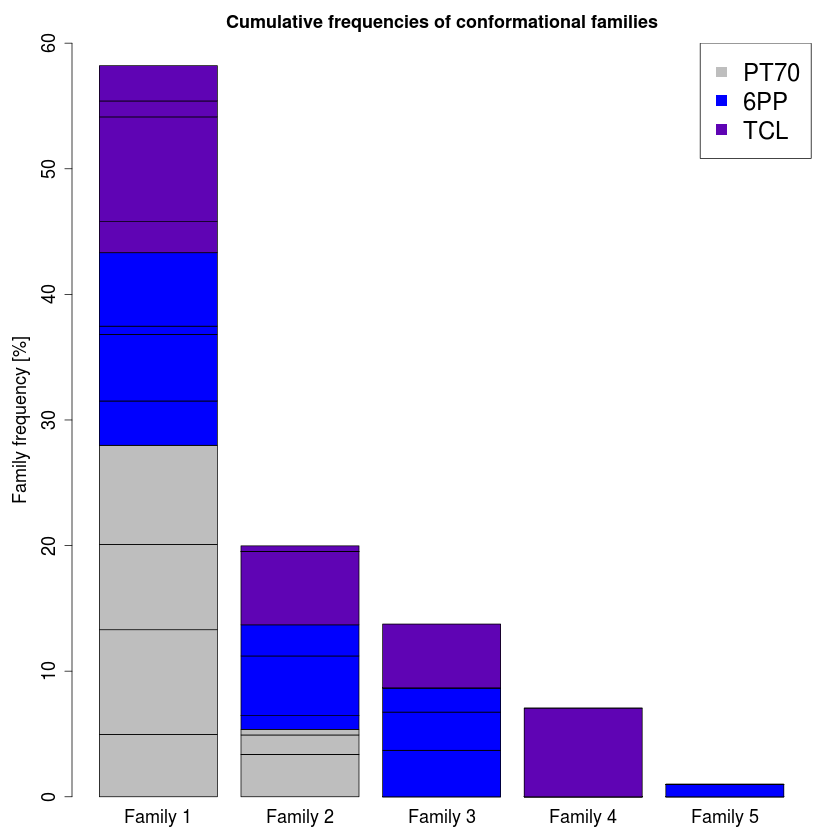

Supplement: S3 Fig — Horizontal lines separate the single monomers of each of the three considered homotetrameric complexes. (TIF) [file pone.0127009.s003.tif]

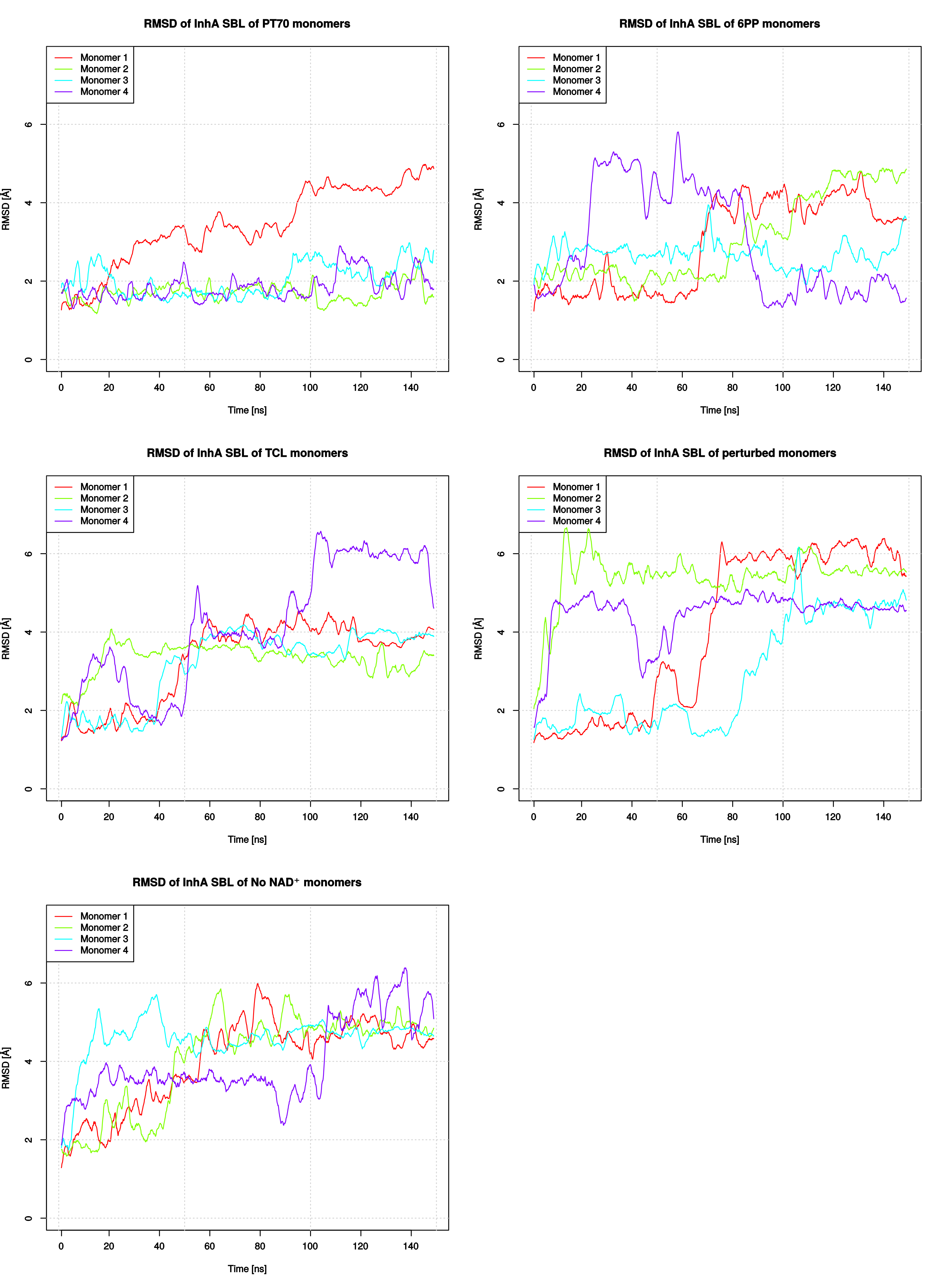

Supplement: S4 Fig — A moving average with a window size of 20 frames was used. The RMSD was measured with reference to chain A of the 2X23 crystal structure. (TIF) [file pone.0127009.s004.tif]

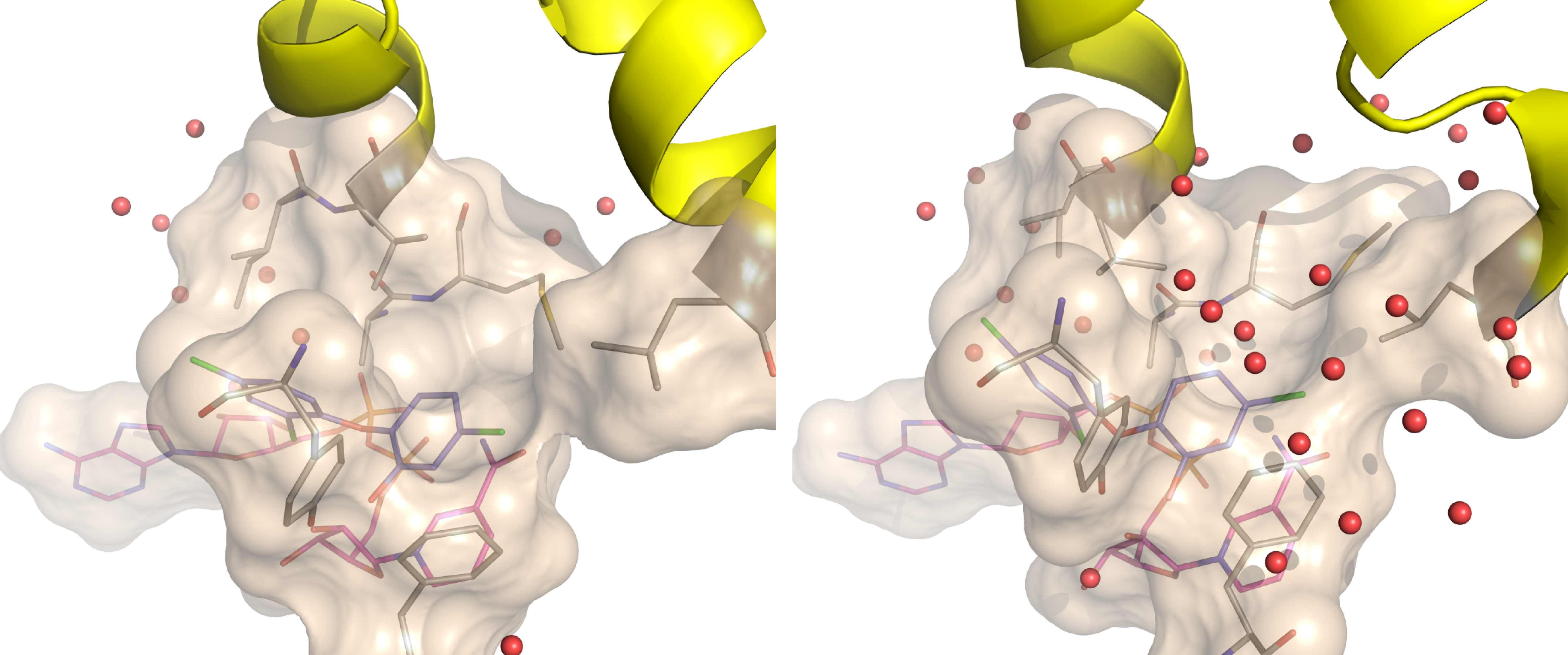

Supplement: S5 Fig — The ligand TCL is depicted in slate blue, the cofactor in magenta and the pocket residues including Leu218 in gray. The SBL is shown in yellow. Ligand, cofactor, and pocket residues are also shown as surface (wheat), oxygens of water molecules are shown in red. Flooding of the hydrophobic pocket is noticeable after 700 ps (right). (TIF) [file pone.0127009.s005.tif]

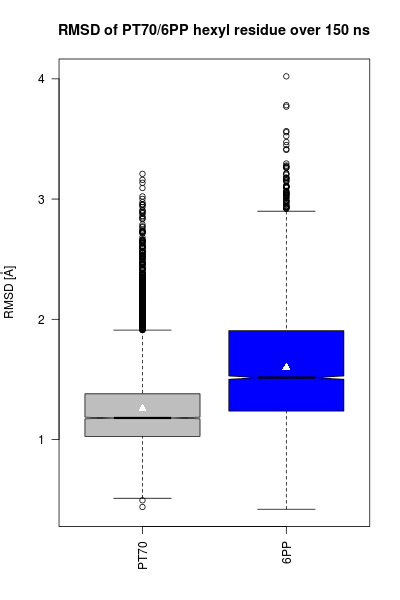

Supplement: S6 Fig — As references the respective coordinates of the starting structure (after the heating cycles) were used (cf. Fig 4 for further explanations). (TIF) [file pone.0127009.s006.tif]

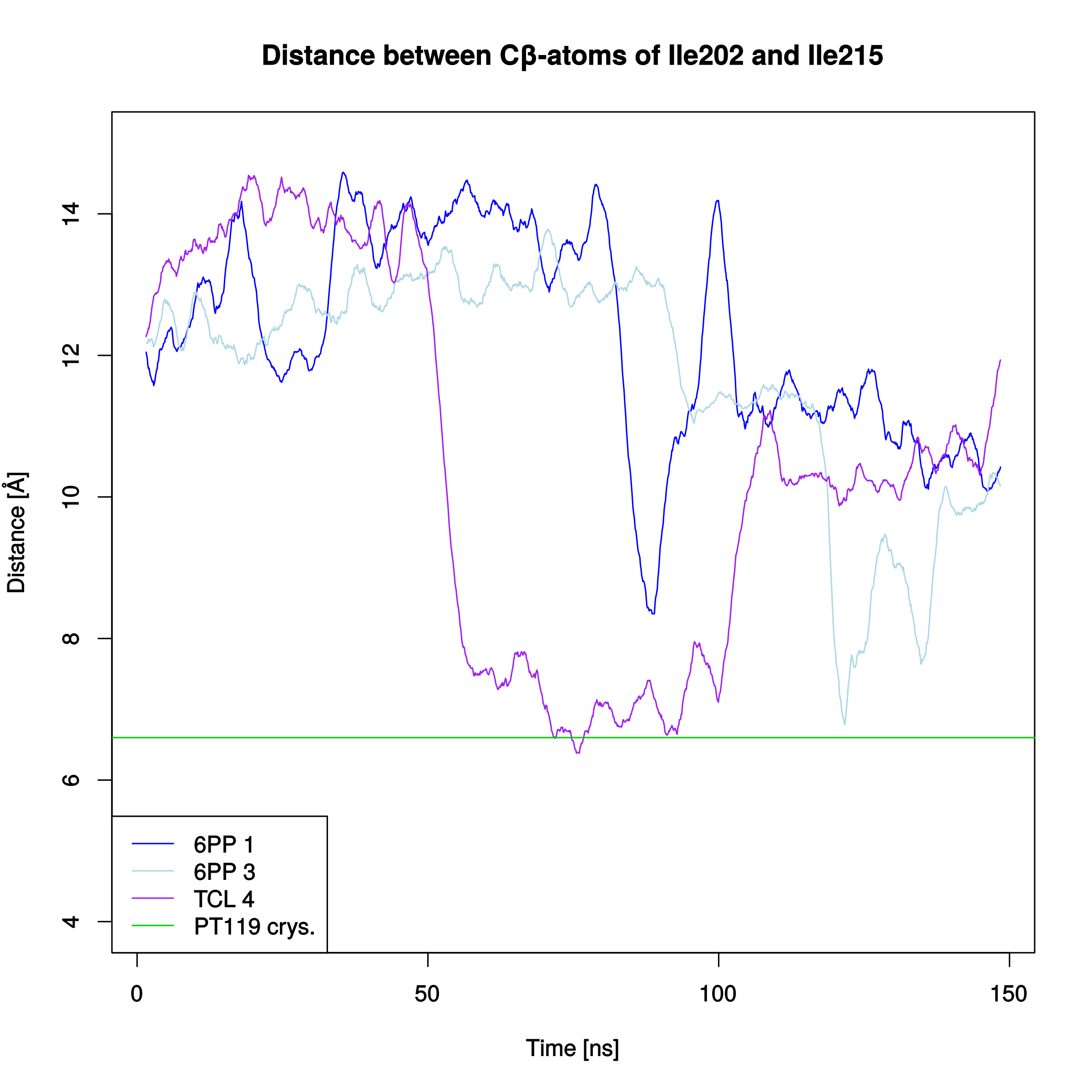

Supplement: S7 Fig — 6PP monomers 1 and 3 are shown in shades of blue, TCL monomer 4 is depicted in purple. The green baseline illustrates the PT119 crystal structure (PDB 4OIM). (TIF) [file pone.0127009.s007.tif]

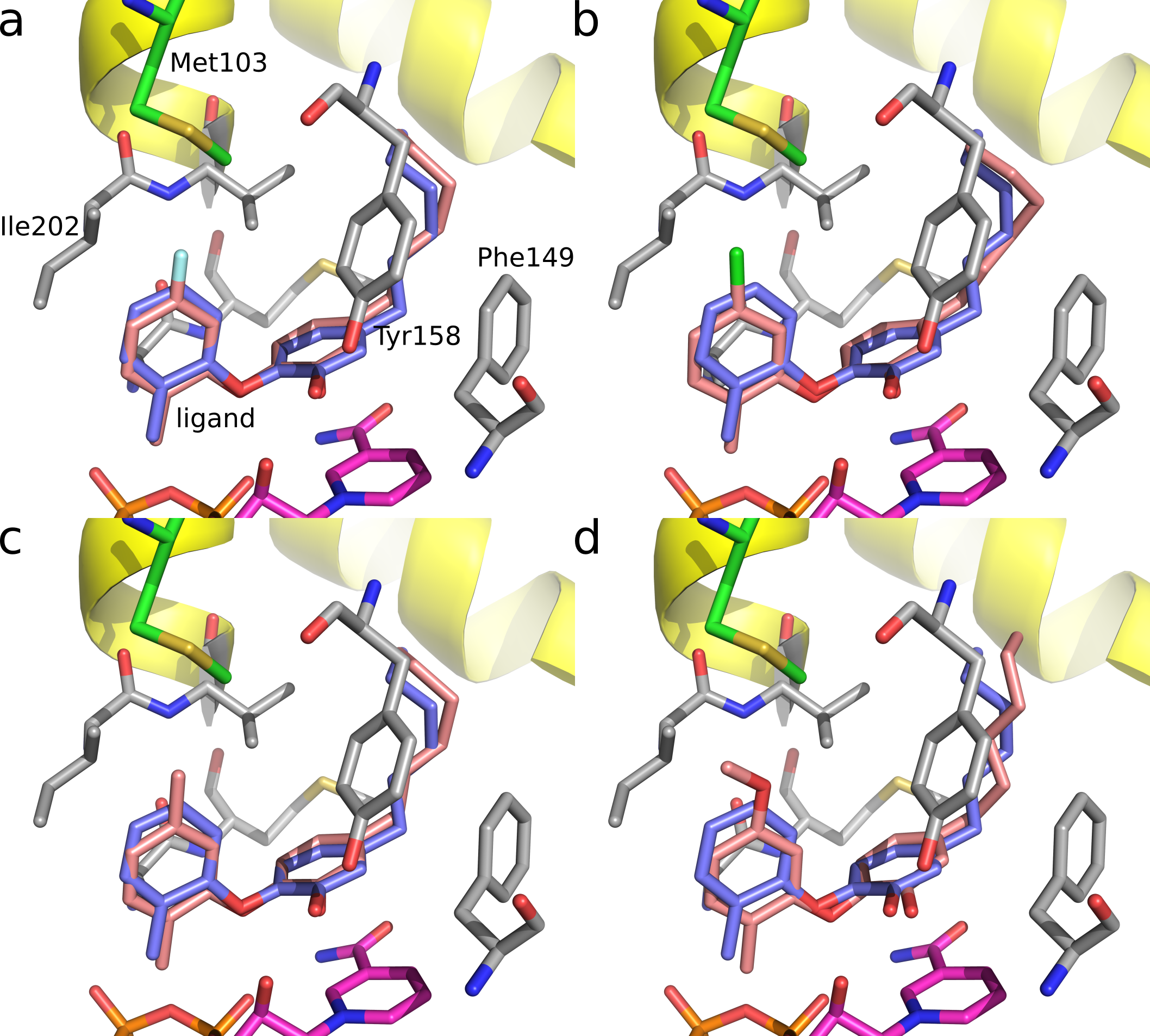

Supplement: S8 Fig — Met103 is illustrated in green, the docked ligands are shown in salmon. PT70 (shown in slate blue as reference) was substituted in 5’-position with a (a) fluoro-, (b) chloro-, (c) methyl-, and (d) methoxy-substituent. Docking was carried out with Glide in XP-mode using default settings and a maximum output of 10 poses per ligand. (TIF) [file pone.0127009.s008.tif]
